# Supplementary material for: DNA Methylation Directs Polycomb-Dependent 3D Genome Re-organization in Naive Pluripotency
Source: Cell Rep. 2019 Nov 12;29(7):1974–1985.e6. doi: 10.1016/j.celrep.2019.10.031 (PMC6856714; doi:10.1016/j.celrep.2019.10.031)
Supplement: Document S1. Figures S1–S5 and Tables S1–S4 [file mmc1.pdf]

Cell Reports, Volume *29*

Supplemental Information

DNA Methylation Directs Polycomb-Dependent 3D Genome Re-organization in Naive Pluripotency

Katy McLaughlin, Ilya M. Flyamer, John P. Thomson, Heidi K. Mjoseng, Ruchi Shukla, Iain Williamson, Graeme R. Grimes, Robert S. Illingworth, Ian R. Adams, Sari Pennings, Richard R. Meehan, and Wendy A. Bickmore

Supplemental Data

Table S1. Inter-probe distances. Related to Figs 1, 2, S1 and S4

Median inter-probe distances for each FISH probe set in all cell lines and conditions. Data from replicate experiments are indicated

Cell line/condition	Probe pair	Median interprobe distance (μm)
**Figures 1 & S1**		
WT (clone36) Serum	*Hoxd3-Hoxd13*	Rep1: 0.276, Rep2: 0.317
WT (clone36) 2i	*Hoxd3-Hoxd13*	Rep1: 0.424, Rep2: 0.423
WT (clone36) Serum	*GCR-Lnp*	Rep1: 0.334, Rep2: 0.36
WT (clone36) 2i	*GCR-Lnp*	Rep1: 0.36, Rep2: 0.422
Ring1B$^{-/-}$ Serum	*Hoxd3-Hoxd13*	Rep1: 0.379, Rep2: 0.443
Ring1B$^{-/-}$ 2i	*Hoxd3-Hoxd13*	Rep1: 0.483, Rep2: 0.459
Ring1B$^{-/-}$ Serum	*GCR-Lnp*	Rep1: 0.3, Rep2: 0.36
Ring1B$^{-/-}$ 2i	*GCR-Lnp*	Rep1: 0.3, Rep2: 0.334
Eed$^{-/-}$ Serum	*Hoxd3-Hoxd13*	Rep1: 0.483, Rep2: 0.481
Eed$^{-/-}$ 2i	*Hoxd3-Hoxd13*	Rep1: 0.39, Rep2: 0.469
Eed$^{-/-}$ Serum	*GCR-Lnp*	Rep1: 0.334, Rep2: 0.334
Eed$^{-/-}$ 2i	*GCR-Lnp*	Rep1: 0.36, Rep2: 0.334
E14 Serum	*Hoxb1-Hoxb13*	0.276
E14 2i	*Hoxb1-Hoxb13*	0.347
E14 Serum	*Hoxc4-Hoxc13*	0.3
E14 2i	*Hoxc4-Hoxc13*	0.36
**Figure 2**		
Blastocysts	*Hoxd3-Hoxd13*	0.422
Blastocysts	*GCR-Lnp*	0.334
**Figure 5 & S4**		
WT J1 Serum	*Hoxd3-Hoxd13*	0.3
WT J1 2i	*Hoxd3-Hoxd13*	0.443
WT J1 Serum	*GCR-Lnp*	0.36
WT J1 2i	*GCR-Lnp*	0.334
3B3L Serum	*Hoxd3-Hoxd13*	Rep1: 0.3, Rep2: 0.3
3B3L 2i	*Hoxd3-Hoxd13*	Rep1: 0.334, Rep2: 0.334
3B3L Serum	*GCR-Lnp*	0.3
3B3L 2i	*GCR-Lnp*	0.334
3A3L Serum	*Hoxd3-Hoxd13*	0.3
3A3L 2i	*Hoxd3-Hoxd13*	0.36
WT J1 Serum	*En2-Shh*	0.324
WT J1 2i	*En2-Shh*	0.385
WT J1 Serum	*Shh-Mnx1*	0.48
WT J1 2i	*Shh-Mnx1*	0.608
WT J1 Serum	*En2-Mnx1*	0.478
WT J1 2i	*En2-Mnx1*	0.59
3B3L Serum	*En2-Shh*	0.329
3B3L 2i	*En2-Shh*	0.247
3B3L Serum	*Shh-Mnx1*	0.44
3B3L 2i	*Shh-Mnx1*	0.44
3B3L Serum	*En2-Mnx1*	0.471
3B3L 2i	*En2-Mnx1*	0.466

Table S2. Related to Figs 1, 2, 5, S1 and S4

Probability values calculated by Mann-Whitney U tests comparing inter-probe distances between two populations. Inter-probe distances of *HoxD*, *Lnp-GCR* (Ctrl), *HoxC*, and *HoxB* probes in different cell types and conditions are shown

Genotype/ Condition/ Probes Sample 1	Genotype/ Condition/ Probes Sample 2	P-value (Mann-Whitney U Test)
**Figure 1 & Figure S1**		
WT (clone36) Serum HoxD	WT (clone36) 2i *HoxD*	Rep1: <0.0001, Rep2: 0.0003
WT (clone36) Serum HoxD	Ring1B $^{-/-}$ Serum *HoxD*	Rep1: <0.0001, Rep2: <0.0001
WT (clone36) Serum HoxD	Eed $^{-/-}$ Serum *HoxD*	Rep1: <0.0001, Rep2: <0.0001
WT (clone36) Serum HoxD	Ring1B $^{-/-}$ 2i *HoxD*	Rep1: <0.0001, Rep2: <0.0001
WT (clone36) Serum HoxD	Eed $^{-/-}$ 2i *HoxD*	Rep1: <0.0001, Rep2: <0.0001
WT (clone36) Serum Ctrl	WT (clone36) 2i Ctrl	Rep1: 0.4215, Rep2: 0.2564
WT (clone36) Serum Ctrl	Ring1B $^{-/-}$ Serum Ctrl	Rep1: 0.1352, Rep2: 0.5583
WT (clone36) Serum Ctrl	Eed $^{-/-}$ Serum Ctrl	Rep1: 0.7539, Rep2: 0.4776
WT (clone36) Serum Ctrl	Ring1B $^{-/-}$ 2i Ctrl	Rep1: 0.0797, Rep2: 0.1836
WT (clone36) Serum Ctrl	Eed $^{-/-}$ 2i Ctrl	Rep1: 0.5062, Rep2: 0.0865
E14 Serum *HoxB*	E14 2i *HoxB*	0.0334
E14 Serum *HoxC*	E14 2i *HoxC*	0.0024
**Figure 2**		
WT serum *HoxD*	Blastocysts *HoxD* (all)	<0.0001
WT serum *HoxD*	Blastocyst 1 *HoxD*	0.0050
WT serum *HoxD*	Blastocyst 2 *HoxD*	0.0170
WT serum *HoxD*	Blastocyst 3 *HoxD*	0.0168
WT serum *HoxD*	Blastocyst 4 *HoxD*	0.0043
WT serum *HoxD*	Blastocyst 5 *HoxD*	<0.0001
WT serum *HoxD*	Blastocyst 6 *HoxD*	<0.0001
WT serum *HoxD*	Blastocyst 7 *HoxD*	0.0068
WT serum *HoxD*	Blastocyst 8 *HoxD*	0.0002
WT serum *HoxD*	Blastocyst 9 *HoxD*	0.0019
WT serum *HoxD*	Blastocyst 10 *HoxD*	0.1389
WT serum *HoxD*	Blastocyst 11 *HoxD*	0.0537
WT serum *HoxD*	Blastocyst 12 *HoxD*	0.0047
WT serum *HoxD*	Blastocyst 13 *HoxD*	0.0237
WT 2i *HoxD*	Blastocysts *HoxD* (all)	0.0879
WT Serum Ctrl	Blastocysts Ctrl	0.6855
WT 2i Ctrl	Blastocysts Ctrl	0.2017
**Figure 5 and Figure S4**		
WT J1 Serum *HoxD*	WT J1 2i *HoxD*	Rep1: <0.0001, Rep2:
WT J1 Serum *HoxD*	3B3L Serum *HoxD*	Rep1: 0.2027, Rep2:
WT J1 Serum *HoxD*	3B3L 2i *HoxD*	Rep1: 0.2790, Rep2:
3B3L Serum *HoxD*	3B3L 2i *HoxD*	Rep1: 0.8658 Rep2:
WT J1 Serum *HoxD*	3A3L Serum *HoxD*	0.7219
WT J1 Serum *HoxD*	3A3L 2i *HoxD*	0.1128
3A3L Serum *HoxD*	3A3L 2i *HoxD*	0.2779
WT J1 Serum Ctrl	WT J1 2i Ctrl	0.2195
WT J1 Serum Ctrl	3B3L Serum Ctrl	0.0513
WT J1 Serum Ctrl	3B3L 2i Ctrl	0.1445
3B3L Serum Ctrl	3B3L 2i Ctrl	0.5587
WT J1 Serum *Shh-Mnx1*	WT J1 2i *Shh-Mnx1*	0.0001
WT J1 Serum *Shh-Mnx1*	3B3L Serum *Shh-Mnx1*	0.2274
WT J1 2i *Shh-Mnx1*	3B3L 2i *Shh-Mnx1*	<0.0001

3B3L Serum *Shh-Mnx1*	3B3L 2i *Shh-Mnx1*	0.3214
WT J1 Serum *En2-Shh*	WT J1 2i *En2-Shh*	0.0228
WT J1 Serum *En2-Shh*	3B3L Serum *En2-Shh*	0.4785
WT J1 2i *En2-Shh*	3B3L 2i *En2-Shh*	<0.0001
3B3L Serum *En2-Shh*	3B3L 2i *En2-Shh*	0.0028
WT J1 Serum *En2-Mnx1*	WT J1 2i *En2-Mnx1*	0.0101
WT J1 Serum *En2-Mnx1*	3B3L Serum *En2-Mnx1*	0.3550
WT J1 2i *En2-Mnx1*	3B3L 2i *En2-Mnx1*	0.0001
3B3L Serum *En2-Mnx1*	3B3L 2i *En2-Mnx1*	0.3190

<u>Table S3. Details of FISH probes. Related to STAR methods</u>

Genome co-ordinates are given using the mm9 assembly of the mouse genome

Locus	Whitehead Name	Coordinates (mm9)	Size (bp)
Hoxd3	WI1-121N10	Chr2: 74,566,983 – 74,605,438	38,455
Hoxd13	WI1-469P2	Chr2: 74,474,157 -74,513,003	38,846
GCR	WI1-2157A11	Chr2: 74,242,615 -74,282,044	39,429
Lnp	WI1-482L15	Chr2: 74,329,582 -74,372,986	43,404
Hoxb1	WI1-2671L18	Chr11: 96,201,164- 96,242,956	41,793
Hoxb13	WI1-1356F15	Chr11: 96,060,900-96,099,631	38,732
Hoxc4	WI1-0991J24	Chr15: 103,018,285 -103,057,123	38,838
Hoxc13	WI1-1176M4	Chr15: 102,910,943 - 102,949,345	38,403
En2	WI1-2728F4	Chr5: 28,477,913 – 28,517,563	39,650
Shh	WI1-574O18	Chr5: 28,754,458 – 28,795,879	41,421
Mnx1	WI1-1204B6	Chr5: 29,791,124 – 29,827,491	36,367

<u>Table S4. Details of Hi-C reads. Related to STAR methods</u>

Details of unmapped and mapped Hi-C sequence reads and cis vs trans contacts for biological replicates of serum and 2i ESCs.

	serum-1	2i-1	serum-2	2i-2	*serum (1+2)*	2i (1+2)
total	395761788	705371075	583052986	463486298	*978814774*	1168857373
total_unmapped	34422169	61538050	47293653	51315320	*81715822*	112853370
total_singlesided_mapped	84677482	119074965	113483585	97305872	*198161067*	216380837
total_mapped	276662137	524758060	422275748	314865106	*698937885*	839623166
total_dups	153386711	88829285	81632665	73187613	*235019376*	162016898
total_nodups	123275426	435928775	340643083	241677493	*463918509*	677606268
cis	106081324	352699387	293259179	209065335	*399340503*	561764722
trans	17194102	83229388	47383904	32612158	*64578006*	115841546

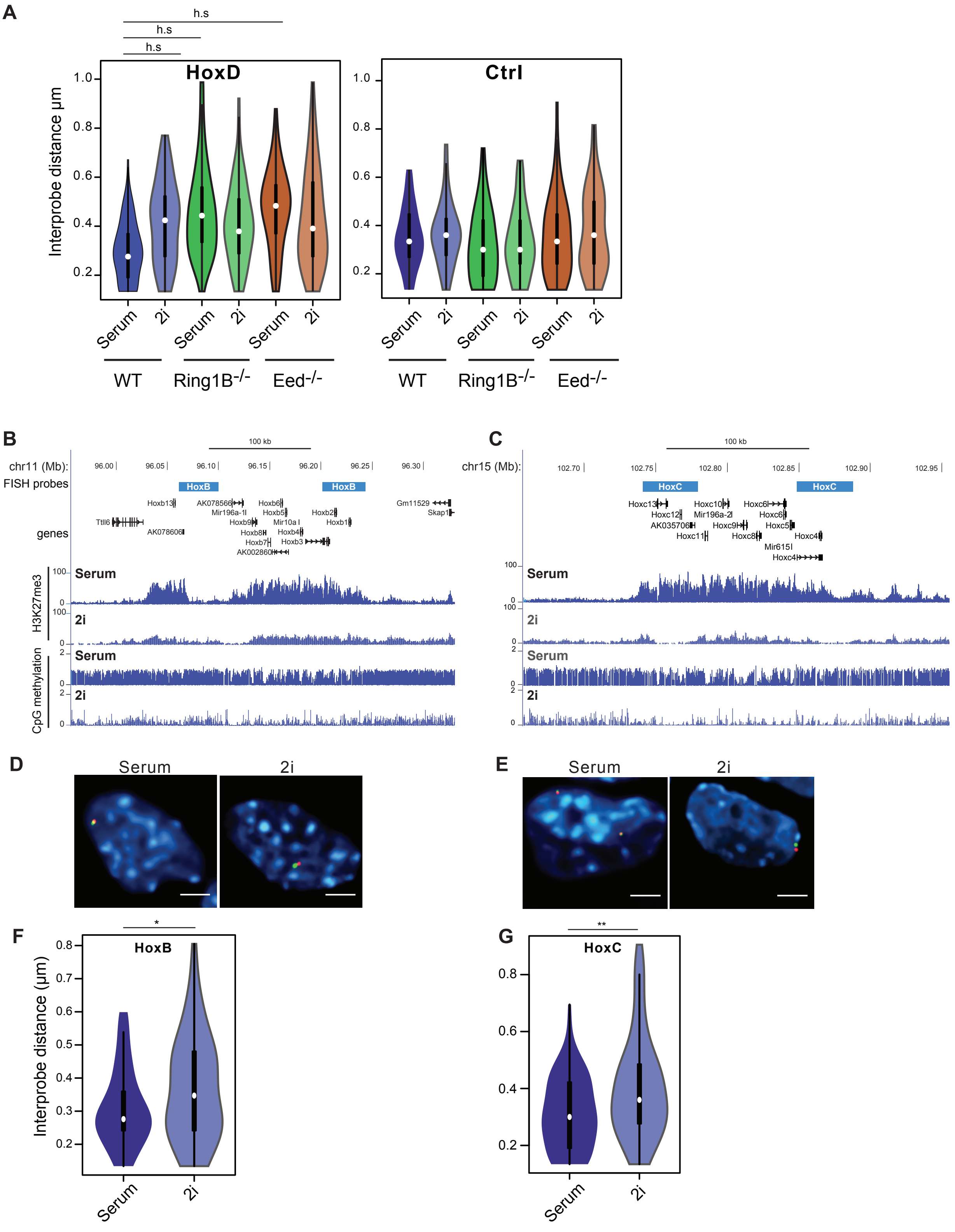

Figure S1. Related to Figure 1.

A. Violin plots showing the distribution of inter-probe distances for HoxD and control (Ctrl) loci (from Figure 1A) in WT, Ring1B-/- and Eed-/- cells grown in serum or 2i. Data are a biological replicate for the data in Figure 1. h.s = p<0.0001. Details of statistical analysis are given in Tables S1 and S2.

B. UCSC genome browser tracks (mm9 assembly of the mouse genome) showing the location on chromosome 11 of FISH probes used to measure compaction across the HoxB locus. Probe co-ordinates are given in Table S3. Below are shown the H3K27me3 (Marks et al., 2012) and DNA methylation (Habibi et al., 2013) profiles for this region of the mouse genome in mESCs grown in serum or 2i.

C. As in (B) but for the HoxC locus on chromosome 15.

D. Representative images of HoxB probe hybridisation signals (red and green) in WT E14 mESCs grown in serum or 2i. Scale bars represent 10 µm.

E. As in (D) but for HoxC.

F. Violin plots showing the distribution of inter-probe distances for the HoxB locus in mESCs cells grown in serum or 2i. The vertical line and spot within each plot indicate the interquartile range and median, respectively. *p<0.05, **p<0.01.

G. As in (F) but for HoxC.

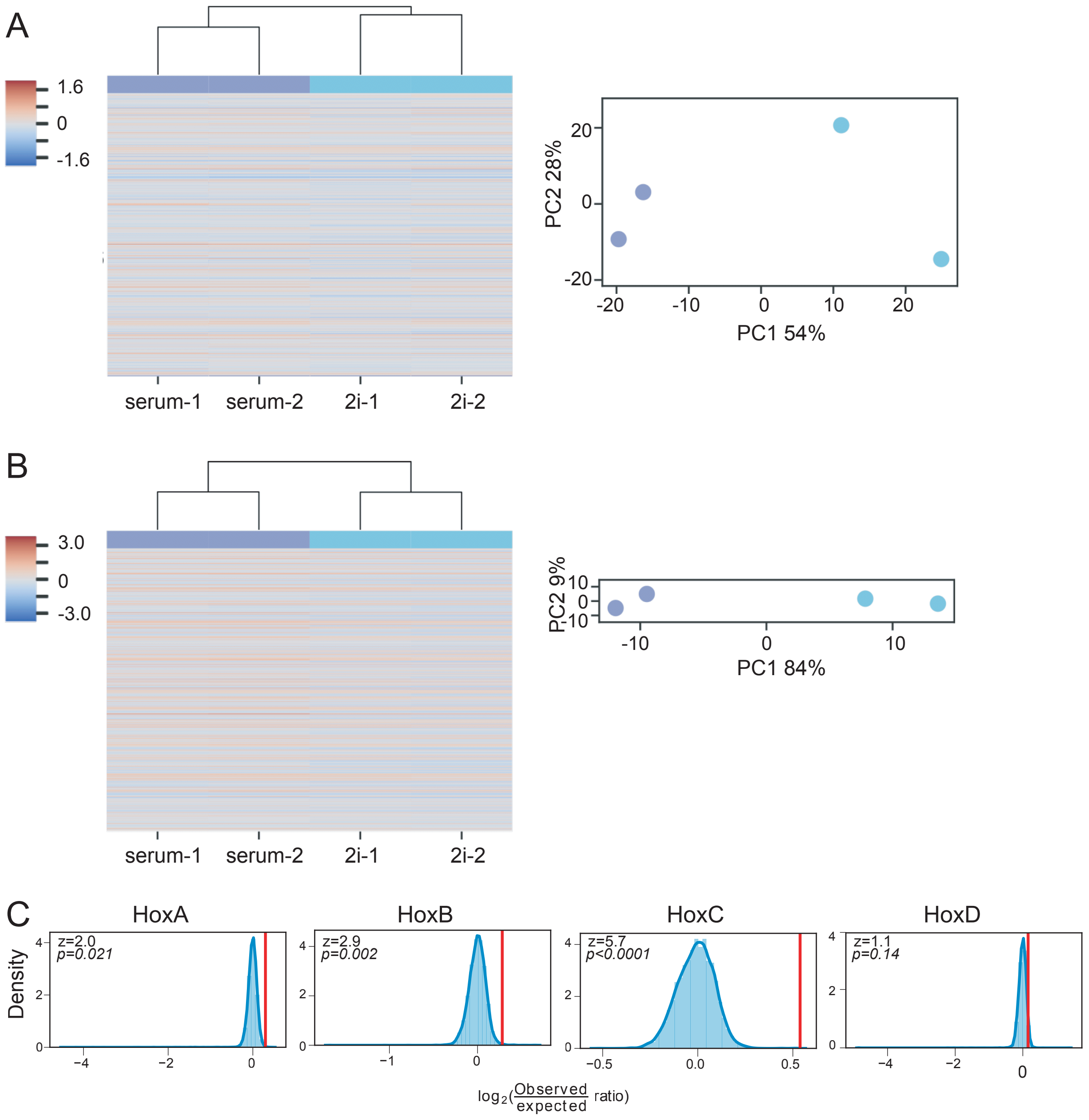

Figure S2. Related to Figure 3.

A. Hierarchical clustering of genome-wide (A) insulation index [25 kb resolution, 1Mb window size] and (**B**) compartment signal/ first eigenvector [200 kb resolution] of Hi-C data from ESCs cultured in serum (purple) or 2i (turquoise). Graphs to the right show principle component analysis of these data.

C. Statistical analysis of the change in number of Hi-C contacts within the four Hox loci. Shown are the distribution of log2(observed/expected) ratios between serum and 2i for 1000 random regions of the same size in the same chromosome as the respective Hox locus; the value for the Hox region is shown with a vertical red line. Z-score and level of significance (p value) are shown in top left corner of each grap

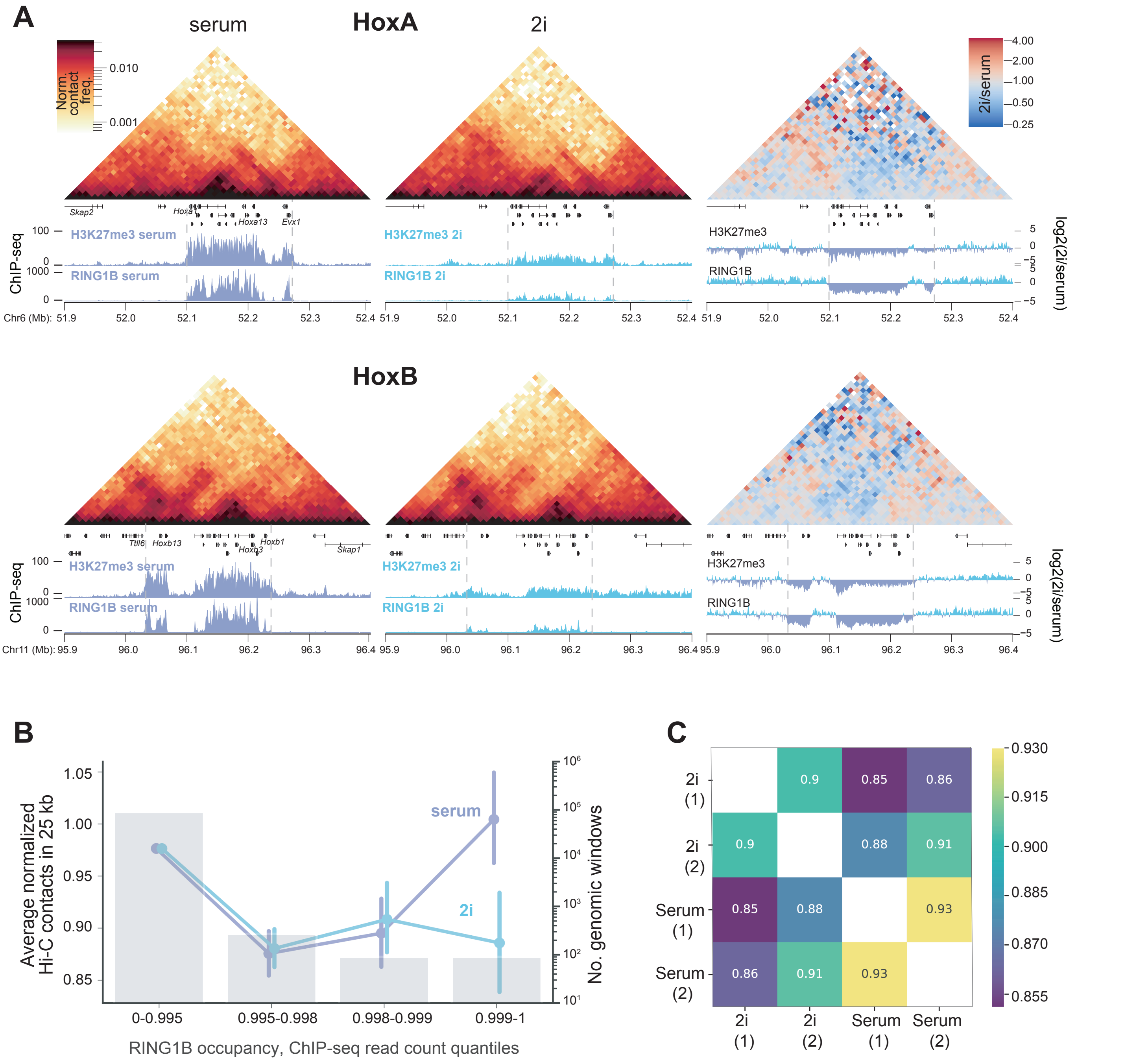

Figure S3. Related to Figure 3.

A. Hi-C heatmaps (normalised contact frequencies) for cells grown in serum (left) and 2i media (middle) for the HoxA (top) and HoxB (bottom). The right hand heatmaps show the difference between contact frequencies in 2i vs serum. Below the gene annotations ChIP-seq profiles for H3K27me3 (Marks et al., 2012), and Ring1B (Joshi et al., 2015) are shown. Boundaries of the Hox clusters are marked with dashed lines. Genome co-ordinates are from mm9 assembly of the mouse genome

B. Same as Fig. 3C, but with RING1B ChIP-seq quantification instead of H3K27me3 (both Hi-C data compared to RING1B data from serum-grown cells). Mean ± 95% CI number of normalized local Hi-C interactions (left hand y axis) in 25 kbp windows across quantiles of RING1B occupancy (Joshi et al., 2015) in serum. Data for serum and 2i media are shown as purple or blue dots respectively. Grey bars show the number of windows in each category (right y axis with log scale).

C. Correlation of log2 of insulation score profiles (100 kb window) across serum and 2i Hi-C replicates.

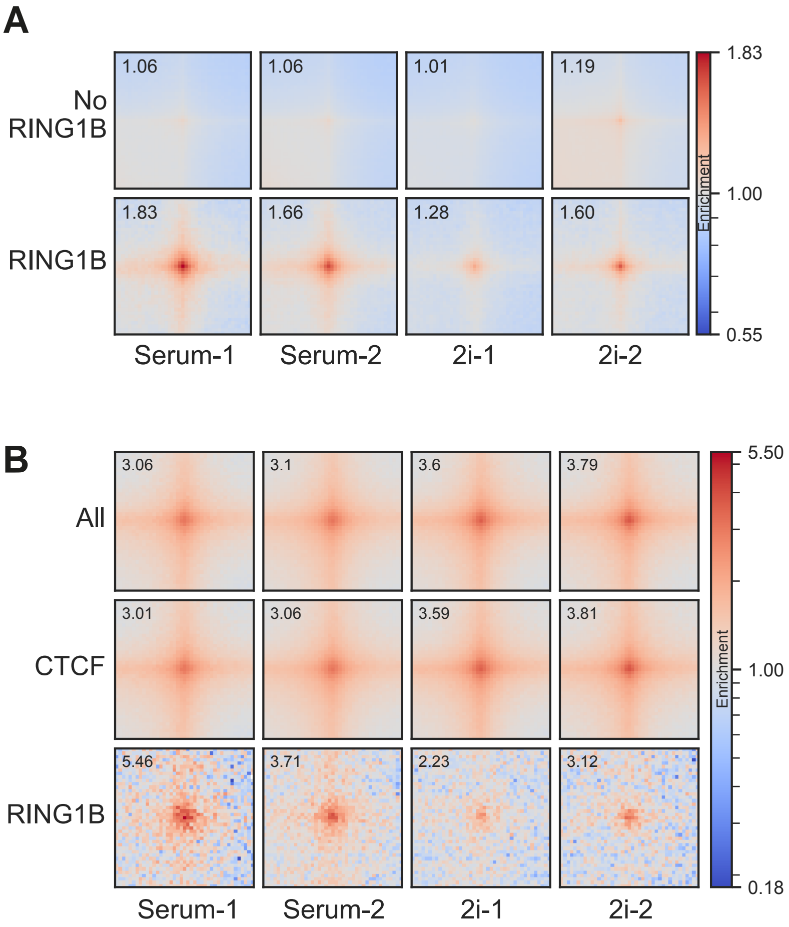

Figure S4. Related to Figure 4.
A. As for main Figure 4C, but for individual replicates.
B. As for main Figure 4D, but for individual replicates of our data.

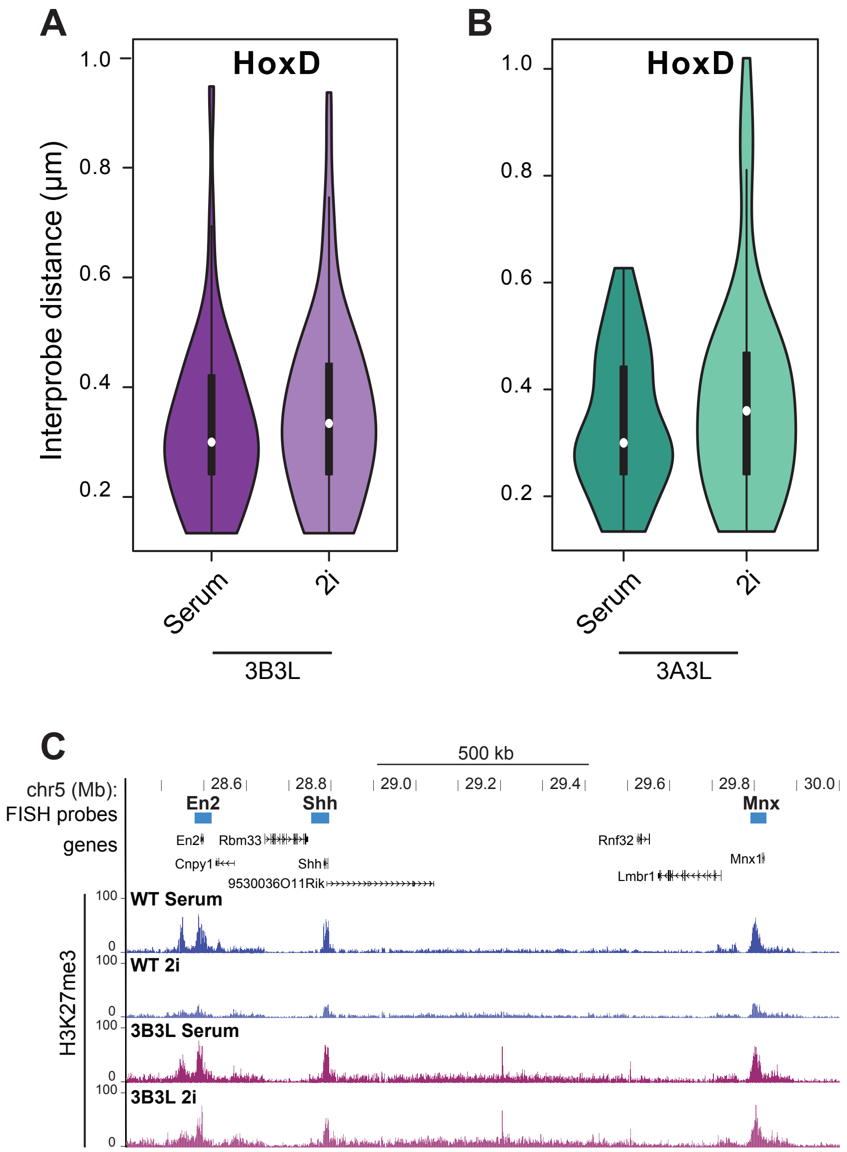

Figure S5. Related to Figure 5.
A. Violin plots showing distribution of inter-probe distances at the HoxD locus in 3B3L cells cultured in serum/LIF and 2i/LIF. This is a biological replicate for the data in Figure 5E.
B. As for (A) but for 3A3L cells.
C. UCSC genome browser tracks (mm9 assembly) showing the location on chromosome 5 of FISH probes used to measure distal interactions across the Shh locus. Probe co-ordinates are given in Table S3. Below are shown the H3K27me3 profiles for this region of the mouse genome in WT (Marks et al., 2012) and 3B3L mESCs grown in serum or 2i.
