# Supplementary material for: DNA Methylation Directs Polycomb-Dependent 3D Genome Re-organization in Naive Pluripotency
Source: Cell Rep. 2019 Nov 12;29(7):1974–1985.e6. doi: 10.1016/j.celrep.2019.10.031 (PMC6856714; doi:10.1016/j.celrep.2019.10.031)
Supplement: Document S2. Article plus Supplemental Information [file mmc2.pdf]

Cell Reports

Article

DNA Methylation Directs Polycomb-Dependent 3D Genome Re-organization in Naive Pluripotency

Graphical Abstract

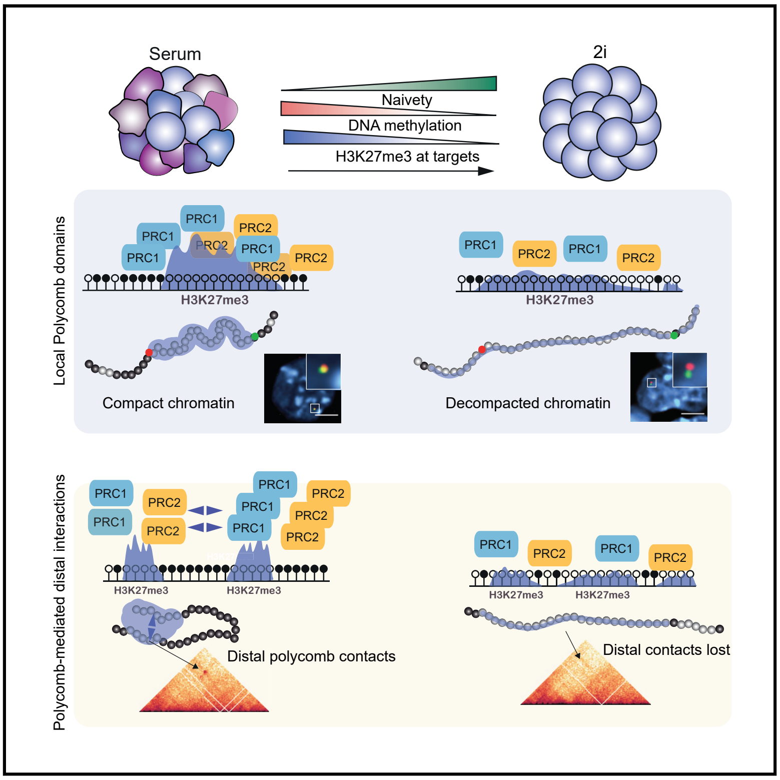

Highlights

- The altered 3D genome of 2i ESCs is due to polycomb re-distribution
- Stopping DNA hypomethylation and genome re-organization does not affect 2i cell state
- Hypomethylated mouse blastocysts have a similar 3D chromatin organization to 2i ESCs

Authors

Katy McLaughlin, Ilya M. Flyamer, John P. Thomson, ..., Sari Pennings, Richard R. Meehan, Wendy A. Bickmore

Correspondence

richard.meehan@igmm.ed.ac.uk (R.R.M.),
wendy.bickmore@igmm.ed.ac.uk (W.A.B.)

In Brief

McLaughlin et al. demonstrate that the global DNA methylation state directs the PRC-dependent 3D organization of mouse ESCs and probably early blastocysts. Their findings highlight a central role for DNA methylation and its influence on polycomb, in shaping major aspects of 3D genome organization in stem cells.

Check for updates

McLaughlin et al., 2019, Cell Reports *29*, 1974–1985
November 12, 2019 © 2019 The Authors.
https://doi.org/10.1016/j.celrep.2019.10.031

CellPress

OPEN ACCESS
CellPress

Cell Reports
Article

DNA Methylation Directs Polycomb-Dependent 3D Genome Re-organization in Naive Pluripotency

Katy McLaughlin,[1,4,6] Ilya M. Flyamer,[1,6] John P. Thomson,[1] Heidi K. Mjoseng,[1] Ruchi Shukla,[1,2] Iain Williamson,[1] Graeme R. Grimes,[1] Robert S. Illingworth,[1,5] Ian R. Adams,[1] Sari Pennings,[3] Richard R. Meehan,[1,7,*] and Wendy A. Bickmore[1,*]
[1]MRC Human Genetics Unit, Institute of Genetics and Molecular Medicine, University of Edinburgh, Crewe Road South, Edinburgh EH4 2XU, UK
[2]Northern Institute for Cancer Research, Framlington Place, Medical Faculty, Newcastle upon Tyne NE2 4HH, UK
[3]Centre for Cardiovascular Science, Queen's Medical Research Institute, University of Edinburgh, 47 Little France Crescent, Edinburgh EH16 4TJ, UK
[4]Present address: Wellcome Centre for Cell Biology, University of Edinburgh, Michael Swann Building, Max Born Crescent, Edinburgh EH9 3BF, UK
[5]Present address: MRC Centre for Regenerative Medicine, Institute for Regeneration and Repair, The University of Edinburgh, Edinburgh BioQuarter, 5 Little France Drive, Edinburgh EH16 4UU, UK
[6]These authors contributed equally
[7]Lead Contact
*Correspondence: richard.meehan@igmm.ed.ac.uk (R.R.M.), wendy.bickmore@igmm.ed.ac.uk (W.A.B.)
https://doi.org/10.1016/j.celrep.2019.10.031

SUMMARY

The DNA hypomethylation that occurs when embryonic stem cells (ESCs) are directed to the ground state of naive pluripotency by culturing in two small molecule inhibitors (2i) results in redistribution of polycomb (H3K27me3) away from its target loci. Here, we demonstrate that 3D genome organization is also altered in 2i, with chromatin decompaction at polycomb target loci and a loss of long-range polycomb interactions. By preventing DNA hypomethylation during the transition to the ground state, we are able to restore to ESC in 2i the H3K27me3 distribution, as well as polycomb-mediated 3D genome organization that is characteristic of primed ESCs grown in serum. However, these cells retain the functional characteristics of 2i ground-state ESCs. Our findings demonstrate the central role of DNA methylation in shaping major aspects of 3D genome organization but caution against assuming causal roles for the epigenome and 3D genome in gene regulation and function in ESCs.

INTRODUCTION

The extent to which epigenetic modifications and three-dimensional (3D) chromatin structure are linked and contribute to cell state and cell function is unresolved. Two key and inter-related epigenetic modifiers in the mammalian genome are DNA methylation and polycomb. Polycomb complexes are implicated in the maintenance of repression of key developmental genes (Blackledge et al., 2015). Whereas polycomb repressive complex PRC2 deposits H3K27me3, the canonical PRC1 complex promotes compact local chromatin structures and longer-range chromatin interactions (Boettiger et al., 2016; Eskeland et al., 2010; Joshi et al., 2015; Kundu et al., 2017; Schoenfelder et al., 2015; Williamson et al., 2012). Chromatin compaction and developmental gene repression are independent of the E3 ligase catalytic activity of Ring1B in canonical PRC1 (Cohen et al., 2018; Eskeland et al., 2010; Illingworth et al., 2015; Kundu et al., 2017; Williamson et al., 2014).

In mammalian cells, the polycomb system is primarily targeted to the unmethylated CpG islands (CGIs) of non- or weakly expressed genes (Blackledge et al., 2015; Li et al., 2017; Riising et al., 2014). Consistent with this, loss of DNA methylation—by exposing new CpG sites—leads to a redistribution of H3K27me3, to satellite and dispersed repeat sequences, while titrating it away from its normal CGI targets (Brinkman et al., 2012; Jermann et al., 2014; Reddington et al., 2013, 2014). This is consistent with a model in which PRC2 can associate transiently and weakly with a large fraction of the genome (Schuettengruber et al., 2017).

One notable instance in which this occurs is in mouse embryonic stem cells (mESCs) cultured with two small molecule inhibitors of MEK1 and glycogen synthase kinase 3 (GSK3); 2i conditions (Marks et al., 2012). mESCs cultured conventionally in the presence of fetal calf serum and LIF (leukemia inhibitory factor) are functionally heterogeneous, with a fraction of cells resembling a state of "naive pluripotency" with unbiased developmental potential and high expression of pluripotency genes. Other cells in the culture more closely resemble a "primed" state, in which they begin expressing early lineage markers and downregulate pluripotency genes (Canham et al., 2010; Hackett and Surani, 2014; Hayashi et al., 2008; Wongtawan et al., 2011). These two states are metastable, with cells in the population fluctuating between the two. By contrast, culturing mESCs serum free, in the presence of 2i blocks differentiation signals and promotes the pluripotency network, resulting in homogeneous expression of pluripotency factors and reduced

 © 2019 The Authors.
This is an open access article under the CC BY license (http://creativecommons.org/licenses/by/4.0/).

expression of early lineage-specific genes (Morgani et al., 2013; Wray et al., 2011; Ying and Smith, 2017).

The epigenetic properties of 2i-cultured mESCs closely resemble those of cells in the pre-implantation inner cell mass (ICM) of the mouse embryo. This includes global DNA hypomethylation (Ficz et al., 2013; Leitch et al., 2013; Marks et al., 2012; Wray et al., 2011). Expression levels of the *de novo* methyltransferases Dnmt3a, Dnmt3b, and the non-catalytic cofactor Dnmt3l are reduced under 2i conditions. Uhrf1 (a Dnmt1 co-factor) is also downregulated at the protein level (Ficz et al., 2013; Grabole et al., 2013; Graf et al., 2017; Habibi et al., 2013; Leitch et al., 2013; von Meyenn et al., 2016; Yamaji et al., 2013). However, coupling these DNA methylation differences to gene expression changes using triple-knockout (TKO) cells that lack all the active Dnmts reveals that only a small (but significant) proportion of gene expression changes under 2i can be directly attributed to DNA methylation loss (Leitch et al., 2013).

Importantly, although global levels of H3K27me3 are not altered in 2i-cultured cells, there is a marked reduction (up to 75%) of H3K27me3 at polycomb targets, including at the Hox clusters (Marks et al., 2012). This is accompanied by reduced occupancy of Suz12 and Ezh2 (PRC2) and Ring1B (PRC1) (Marks et al., 2012; Joshi et al., 2015). The consequences of such a dramatically altered epigenome on 3D genome organization have not been explored. Given the epigenetic alterations that occur in 2i, and the role of polycomb in shaping the 3D genome, we sought to investigate whether 2i culturing conditions impact on 3D chromatin organization in mESCs. Using fluorescence *in situ* hybridization (FISH) and Hi-C, we show that both local chromatin compaction at polycomb-target Hox loci and long-range polycomb interactions are profoundly altered in 2i, and we demonstrate that this is directly attributable to the loss of DNA methylation. By restoring the epigenetic landscape (DNA methylation and H3K27me3 targeting) of cells in 2i, we show that 3D genome organization can be reset to resemble that of mESCs grown in serum. Strikingly, this has a limited impact on gene expression.

RESULTS

Chromatin Decompaction of Polycomb Target Loci in Naive ESCs

mESCs cultured in a chemically defined medium in the presence of LIF and two inhibitors (2i) of the Erk and Gsk-3 signaling pathways achieve a homogeneous ground state of pluripotency, thought to closely resemble that of the ICM (Ying and Smith, 2017; Ying et al., 2008). In doing so, 2i mESCs acquire a distinct epigenomic landscape, including global DNA hypomethylation and an altered genomic distribution of H3K27me3 (Ficz et al., 2013; Habibi et al., 2013; Leitch et al., 2013; Marks et al., 2012) This includes a loss of H3K27me3 enrichment at classic polycomb targets such as Hox loci (Figure 1A).

Since polycomb is a powerful mediator of higher-order chromatin structure (Boettiger et al., 2016; Eskeland et al., 2010; Francis et al., 2004; Joshi et al., 2015; Kundu et al., 2017; Schoenfelder et al., 2015; Williamson et al., 2012), it is possible that the redistribution of H3K27me3/polycomb across the genome results in an alteration to 3D chromatin organization in ESCs grown in 2i culture conditions, but this has not been investigated.

The murine *HoxD* locus is a large canonical polycomb target in mESCs, demarked by a domain of H3K27me3, PRC2, and PRC1 deposition across the 100-kb cluster (Illingworth et al., 2012). Under serum/LIF culture conditions the *HoxD* locus is maintained in a compact chromatin conformation in mESCs, and this is dependent on the presence of PRC1 (Eskeland et al., 2010; Williamson et al., 2014). To investigate higher-order chromatin compaction at *HoxD* in mESCs grown under serum and 2i conditions, we used 3D FISH to measure the separation of hybridization signals from probe pairs at opposite ends of the *HoxD* locus (*Hoxd3* and *Hoxd13*) under the different conditions. We compared these measurements to those from control probes at a nearby genomic region (3′ of *Lnp*) that is not coated by H3K27me3 but that is highly DNA methylated in serum-grown ESCs (Figure 1A).

Under 2i/LIF culture conditions, the *HoxD* locus significantly decompacts relative to cells cultured in serum/LIF; median inter-probe distances increase from ~300 to ~400 nm, p = < 0.0001 (Figures 1B, 1C, and S1A; Tables S1 and S2). This decompaction occurs to the same extent when either PRC1 (*Ring1B*$^{-/-}$) or PRC2 (*Eed*$^{-/-}$) is absent in mESCs grown under serum conditions (Figures 1B, 1C, and S1A). No further decompaction is observed when PRC1 or PRC2 mutant mESCs are grown under 2i conditions, showing that decompaction of a polycomb target in 2i can be primarily accounted for by the titration of H3K37me3/polycomb away from these genomic regions. We confirmed these data for two other Hox clusters: *HoxB* (Figures S1B, S1D, and S1F) and *HoxC* (Figures S1C, S1E, and S1G).

As a control, we examined a locus not marked by H3K27me3, and highly DNA methylated, in serum-grown ESCs, that is adjacent to *HoxD* (Figure 1A). Inter-probe distances at this control locus were not significantly different between wild-type (WT) or polycomb mutant mESCs, or between mESCs grown in the different culture conditions (Figures 1C and S1A), even though this region is subject to DNA hypomethylation in 2i (Figure 1A). This suggests that the chromatin decompaction we detect in 2i conditions at polycomb target loci is not a result of a general/global alteration in the 3D chromatin organization of naive 2i/LIF cells, and that global loss of DNA methylation across genomic regions may have no direct effect on chromatin compaction, as assayed at a cytological level.

HoxD Chromatin Compaction in the Blastocyst Is Comparable to That in 2i mESCs

Next, we investigated whether the chromatin decompaction observed in 2i-cultured mESCs is also present in the cells of the mouse blastocyst, which are hypomethylated during normal development (Messerschmidt et al., 2014). To compare chromatin states between *in vitro* mESCs and their *in vivo* counterparts, we measured distances between HoxD probes in embryonic day (E) 3.5 mouse blastocysts using 3D FISH (Figure 2A).

These data indicate that the *HoxD* locus in the pre-implantation blastocyst is decompact relative to that in conventionally cultured serum/LIF mESCs, and closely resembles the compaction state of the locus under 2i/LIF conditions (Figures 2B and 2C; Tables S1 and S2). There is a large amount of variability between and within blastocysts, which is likely because these blastocysts will contain distinct cell lineages (trophectoderm, ICM, and

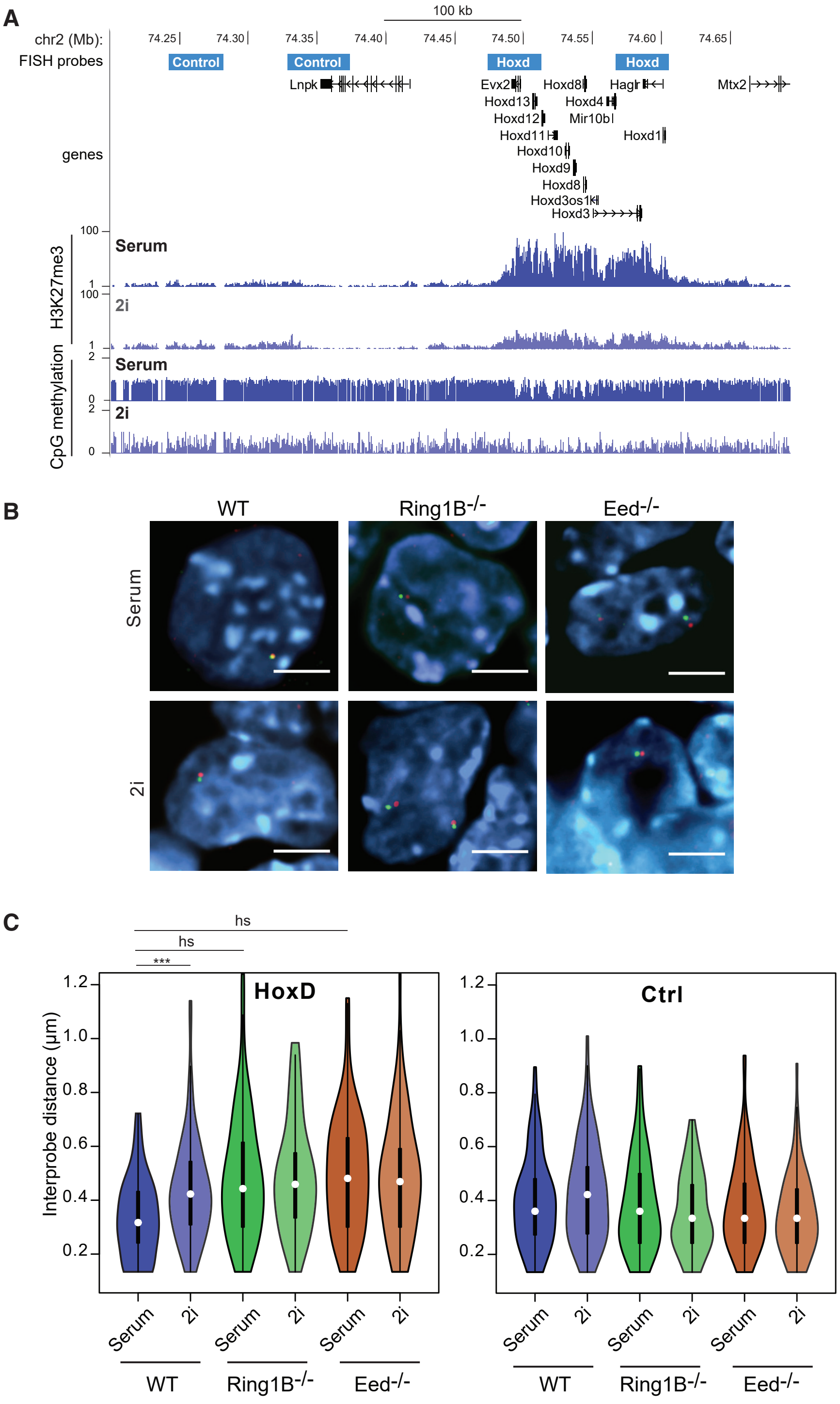

Figure 1. Loss of Chromatin Compaction at Polycomb Target Loci in 2i

(A) UCSC genome browser tracks (mm9 assembly) showing the location (Mb) on chromosome 2 of FISH probes used to measure compaction across the *HoxD* locus, and at a control locus. Probe coordinates are given in Table S3. Below are shown the H3K27me3 ChIP-seq (Marks et al., 2012) and DNA methylation bisulphite (Habibi et al., 2013) profiles for this region of the genome in mESCs grown in serum or 2i.

(B) Representative images of FISH signals (red and green) from probes (indicated in A) detecting the *HoxD* locus in the nuclei of WT, *Ring1B*$^{-/-}$, and *Eed*$^{-/-}$ mESCs. DNA is counterstained with DAPI (blue). Scale bars represent 10 µm.

(C) Violin plots showing the distribution of inter-probe distances (µm) for *HoxD* and control (Ctrl) loci in WT, *Ring1B*$^{-/-}$, and *Eed*$^{-/-}$ cells grown in serum or 2i. The vertical line and spot within each plot indicate the interquartile range and median, respectively.

***$p < 0.001$; h.s., highly significant ($p < 0.0001$).

Full details of statistical analysis are in Tables S1 and S2.

primitive endoderm), all of which are hypomethylated (Rossant et al., 1986). In contrast, inter-probe distances at the control locus were much more similar between blastocysts and cultured cells (Figure 2B), suggesting the decompaction at *HoxD* in the blastocyst cannot be explained by the *in vivo* population having a generally more open chromatin structure.

Altered Local Interactions at Polycomb Loci between Serum and 2i-Cultured mESCs

Polycomb is responsible for forming self-interacting topologically associated domains (TADs) at *Hox* loci as detected by chromosome conformation capture methods (Kundu et al., 2017; Noordermeer et al., 2011; Williamson et al., 2014). To assess whether changes in 3D chromatin organization occur in 2i cells at regions other than *Hox* loci, we employed *in situ* Hi-C (Lieberman-Aiden et al., 2009; Rao et al., 2014) to assay genome-wide chromatin interactions from E14 mESCs grown in serum/LIF and in 2i/LIF, generating two independent Hi-C datasets for each condition. Cluster analysis of local insulation profiles showed separation of the two culture conditions, but the differences were small (Figure S2A). Similar analysis of the eigenvector tracks revealed a larger effect of culture conditions on compartmentalization (Figure S2B).

Inspection of the Hi-C contact frequency heatmaps showed apparent depletion of Hi-C contact frequencies at all four *Hox* loci (*A*, *B*, *C*, and *D*) in cells grown in 2i corresponding to the regions where H3K27me3 and RING1B occupancies are depleted in 2i (Figures 3A and S3A). *Z* score analysis confirms the significant depletion of Hi-C contacts at *Hox*A, -*B*, and -*C* while at *HoxD* the loss of interactions in 2i is not statistically significant (Figure S2C).

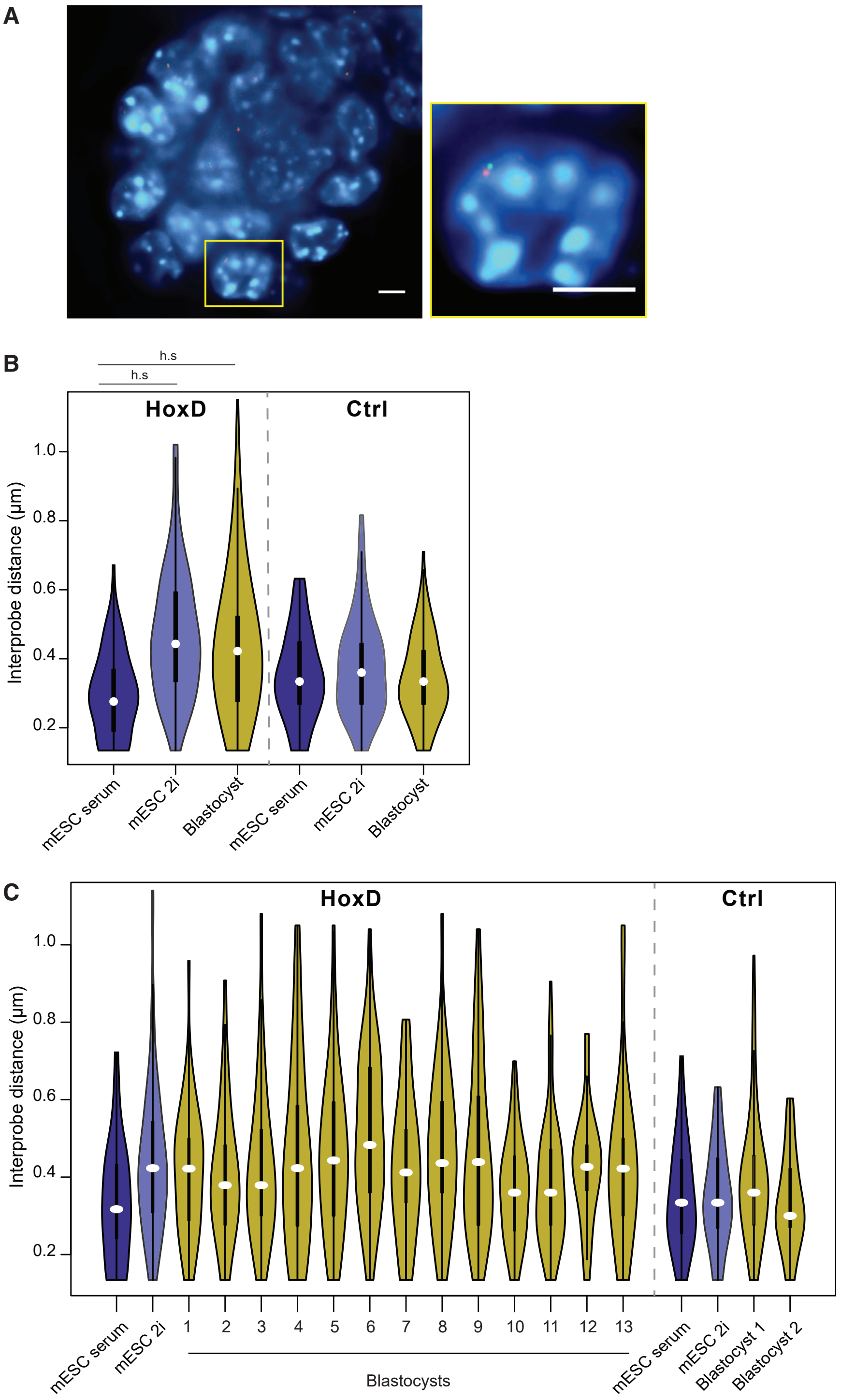

Figure 2. HoxD Chromatin Compaction in the Pre-implantation Blastocyst

(A) Representative image of a DAPI-stained (blue) whole E3.5 blastocyst following FISH with probe pairs (red and green) detecting the *HoxD* locus. Inset shows enlargement of one nucleus. Scale bars represent 10 µm.

(B) Violin plots showing the distribution of interprobe distances (µm) for *HoxD* and control (Ctrl) loci in E14 mESCs grown in serum or 2i, and in E3.5 blastocysts. Data are presented as in Figure 1C. h.s. = p < 0.0001. Full details of statistical analysis are in Tables S1 and S2.

(C) As in (B), but for data from 13 individual blastocysts.

Rescaled pileups of Hi-C data confirmed the enriched contact frequency genome wide for all sites where RING1B occupancy in serum-cultured ESCs occurs over >10 kb (Figure 3B) and that these contacts are greatly depleted in cells grown in 2i culture conditions. Plotting the average number of observed/expected Hi-C contacts in sliding 25-kb windows, split into quantiles by the H3K27me3 chromatin immunoprecipitation sequencing (ChIP-seq) read density in serum (Marks et al., 2012), also showed (Figure 3C) that a high level of polycomb occupancy correlates with high local contact frequency in Hi-C in serum conditions, and that local Hi-C interactions are globally depleted in mESCs that were grown in 2i specifically at the genomic regions most enriched in H3K27me3 in serum conditions.

The same was observed when Hi-C contacts were assessed against RING1B occupancy (Illingworth et al., 2015) (Figure S3B). In contrast, local Hi-C interactions globally are similar between 2i- and serum-grown cells, as assessed by correlation of their insulation scores (Figure S3C), consistent with the preservation of CTCF occupancy reported between serum and 2i-grown mECS (Atlasi et al., 2019).

Loss of Polycomb-Mediated Long-Range Interactions in 2i-Cultured mESCs

Polycomb has also been implicated in more long-range interactions using 4C, 5C, and promoter-capture Hi-C (Bonev et al., 2017; Denholtz et al., 2013; Joshi et al., 2015; Kundu et al., 2017; Schoenfelder et al., 2015; Vieux-Rochas et al., 2015). Visual inspection of our Hi-C data within defined genomic windows in serum/LIF mESCs confirms that there are strong contacts between separate polycomb (H3K27me3) marked loci—for example between the *Skida1* and *Bmi1* loci separated by 650 kb on mouse chromosome 2 (Figure 4A), and between the *En2*, *Shh*, and *Mnx1* loci across 1.3 Mb on chromosome 5 (Figure 4B). Consistent with the redistribution of H3K27me3 and the loss of PRC1 and PRC2 occupancy at these loci under 2i conditions, these long-range

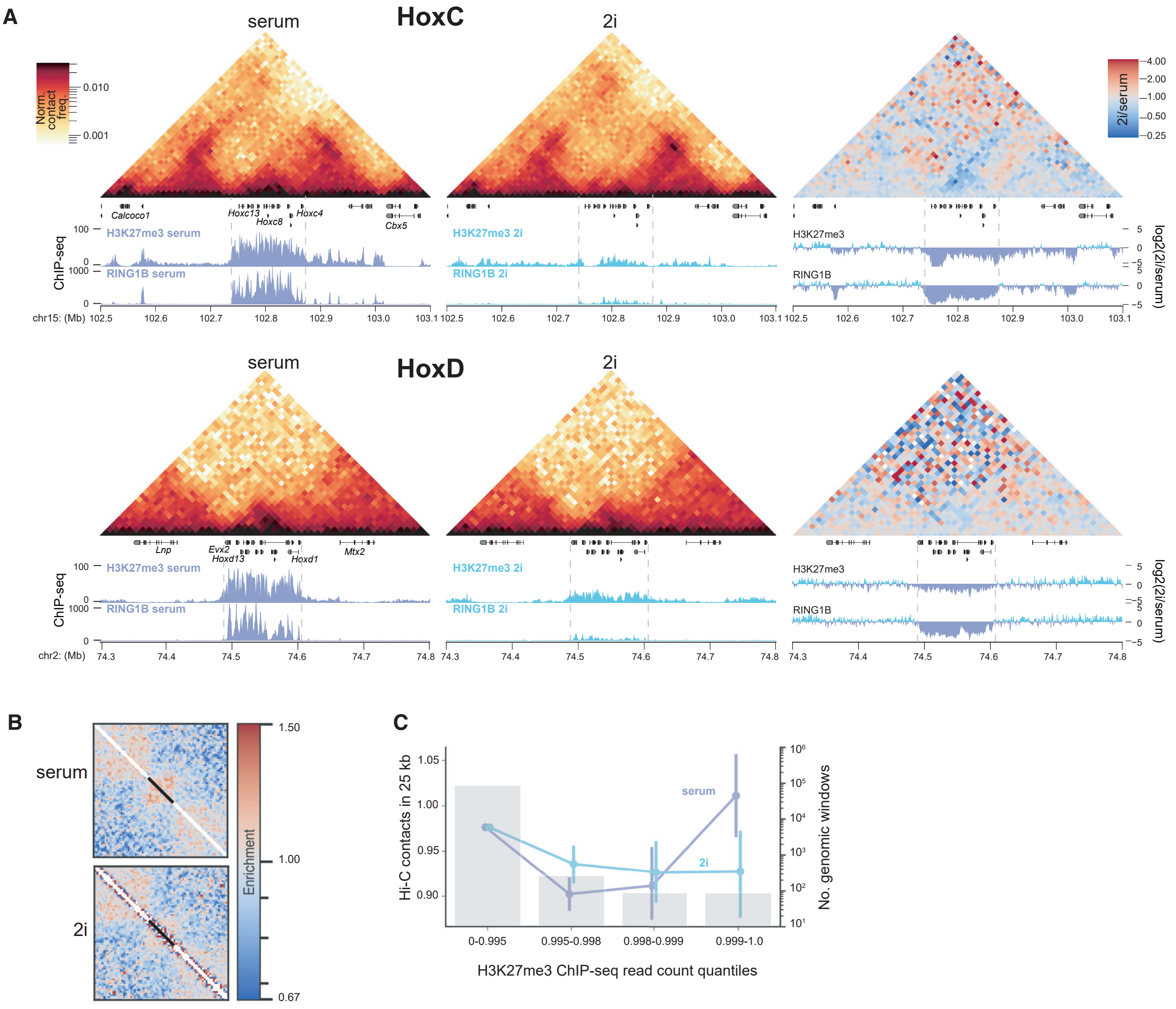

Figure 3. Loss of Local Chromatin Interactions in 2i

(A) Hi-C heatmaps (normalized contact frequencies at 10-kb resolution) for cells grown in serum (left) and 2i media (middle) for the *HoxC* and *HoxD* clusters. The right-hand heatmaps show the difference between contact frequencies in 2i versus serum. Boundaries of the *Hox* clusters are marked with dashed lines. Below the gene annotations, ChIP-seq profiles for H3K27me3 (Marks et al., 2012) and Ring1B (Joshi et al., 2015) are shown. Genome coordinates are from mm9 assembly of the mouse genome

(B) Local rescaled pileups (Flyamer et al., 2019) of all long (> 10 kb) regions of RING1B binding (n = 181) in serum and 2i Hi-C data. Black bar shows the location of the averaged RING1B binding sites.

(C) Mean ± 95% confidence interval (CI) number of normalized local Hi-C interactions (left-hand y axis) in 25-kb windows across quantiles of H3K27me3 occupancy in serum. Data for serum and 2i media are shown as purple or blue dots, respectively. Grey bars show the number of windows in each category (right y axis with log scale).

contacts are depleted or lost from cells in the ground state (Figures 4A and 4B). To analyze such interactions genome wide, we used pileup averaging of intra-chromosomal interactions between all CGIs either bound by PRC1 (RING1B), or not (Figures 4C and S4A). This showed reduced interactions under 2i conditions at CGIs specifically bound by RING1B, suggesting that the interactions disrupted under 2i are related to polycomb and not to general features of CGI promoters. Reduced interactions at polycomb sites were also confirmed by analysis of loops annotated in published Hi-C data from mESCs (Bonev et al., 2017). RING1B-associated loops across the genome display a clear depletion of interactions in 2i cells compared to those grown in serum (Figures 4D and S4B). In contrast, interactions between CTCF sites were not diminished and even seem enhanced in 2i. We also performed the same analysis on published Hi-C data from ICM/E3.5 embryos (Du et al., 2017; Ke et al., 2017; Zhang

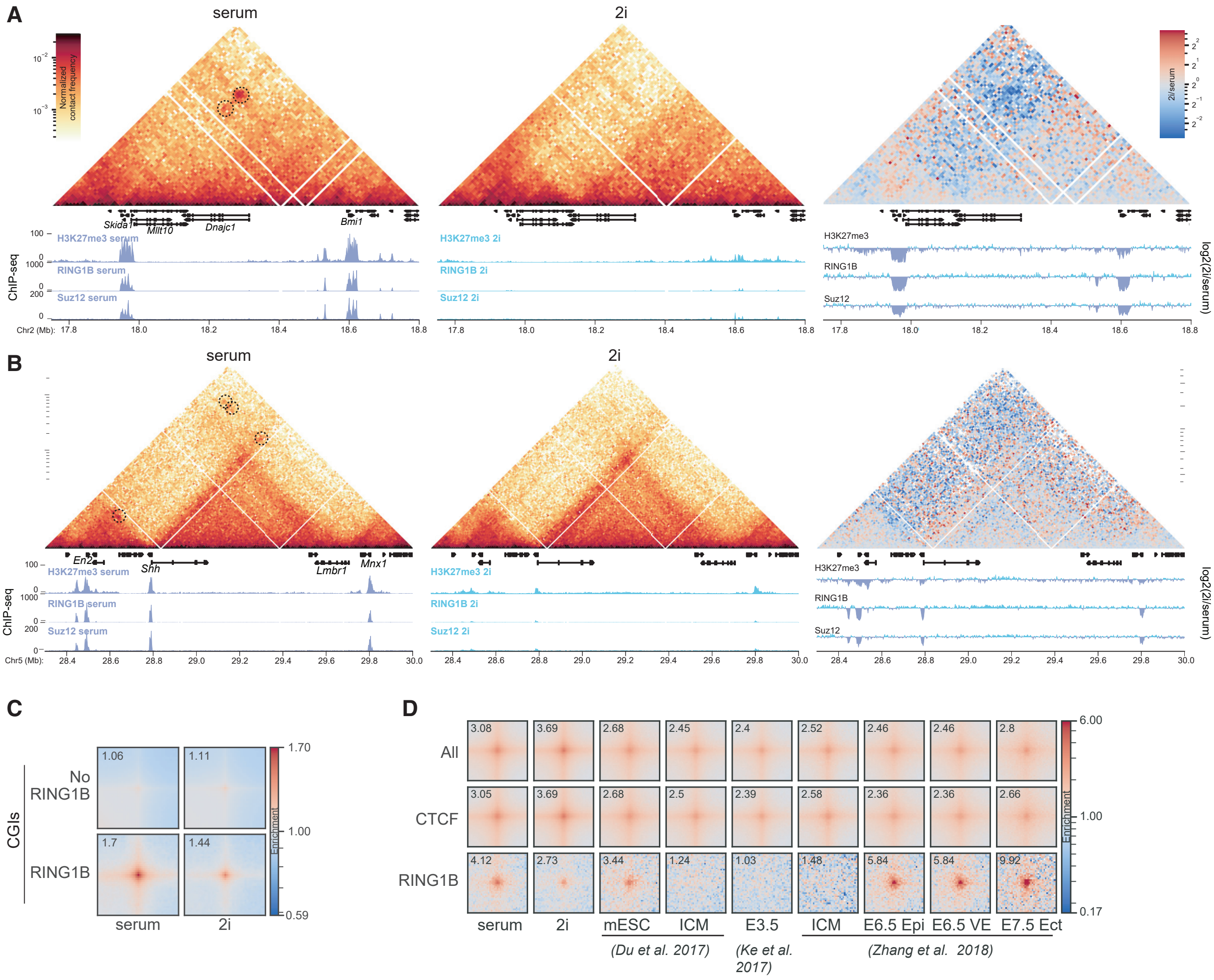

Figure 4. Loss of Long-Range Chromatin Interactions between Polycomb Loci in 2i

(A and B) Hi-C heatmaps (normalized contact frequencies at 10-kb resolution) for cells grown in serum (left) and 2i media (middle) showing distal interactions between polycomb targets, *Bmi1* and *Skida1* (A), or *En2*, *Shh*, and *Mnx1* (B). Interactions in data from serum-cultured cells are highlighted with dashed circles. The right-hand heatmaps show the differences between contact frequencies in 2i versus serum. Below the gene annotations, ChIP-seq profiles for H3K27me3 (Marks et al., 2012), Ring1B, and Suz12 (Joshi et al., 2015) are shown. Genome coordinates are from mm9 assembly of the mouse genome.

(C) Averaged interactions ("pileups") between CpG islands (CGIs) either occupied, or not, by RING1B in Hi-C data from serum- and 2i-cultured cells. Value of the center pixel is shown in the top left corner of each heatmap.

(D) Pileups at loops called in mESC Hi-C data (Bonev et al., 2017), using our serum and 2i Hi-C data, and compared to published Hi-C data from ICM (Du et al., 2017; Zhang et al., 2018), E3.5 embryos (Ke et al., 2017), and from the epiblast (Epi) and visceral endoderm (VE) of the E6.5 embryo and the ectoderm (Ect) at E7.5 (Zhang et al., 2018). Shown are all loops (All), those associated with CTCF peaks but not RING1B peaks (CTCF), and those associated with RING1B peaks (RING1B), but not CTCF peaks. Association is determined by the highest enriched pixel in the loop being within 5 kb of a ChIP-seq peak on both ends, while lack of a peak on at least one of the sides is treated as no association. Value of the center pixel is shown in the top-left corner of each heatmap.

et al., 2018). While we cannot be sure of the polycomb distribution across the genome at this stage of embryogenesis *in vivo*, consistent with a DNA hypomethylated state, we observe high levels of enrichment for CTCF-associated loops in these datasets, but no enrichment at sites corresponding to RING1B-associated loops (Figure 4D). However, enrichment of RING1B-associated loops appears very prominently later in embryogenesis at E6.5 in cells of both the epiblast and visceral endoderm, when DNA methylation is very high (Figure 4D) (Zhang et al., 2018).

Preservation of DNA Methylation in 2i Prevents *HoxD* Decompaction

The 3D chromatin re-organization at polycomb targets we observe under 2i conditions could be a consequence of DNA hypomethylation-mediated polycomb redistribution or a reflection of the altered developmental potential of mESCs cultured in 2i relative to their serum counterparts. To distinguish between these two possibilities, we sought to uncouple the epigenetic transitions from the developmental changes in 2i cells.

DNA hypomethylation in 2i is thought to be the consequence of repression of *Dnmt3a*, *Dnmt3b*, and *Dnmt3l* by PRDM14 (Ficz et al., 2013; Yamaji et al., 2013). Therefore, we established a mESC line in which a high level of DNA methylation is maintained under 2i conditions. This was achieved utilizing a DKO (*Dnmt3a*$^{-/-}$, *Dnmt3b*$^{-/-}$) mESC cell line (3B3) in which DNA methylation is subsequently maintained with a Dnmt3B-expressing transgene under the control of the CAG promoter (Jackson et al., 2004). In addition, we expressed the *de novo* methyltransferase co-factor Dnmt3L from a CAG promoter to create the mESC line 3B3L. Unlike the endogenous gene loci, the *Dnmt3b* and *Dnmt3l* transgenes in 3B3L cells are not repressed by PRDM14. High-performance liquid chromatography (HPLC) confirmed that high CpG DNA methylation levels are retained in 3B3L cells cultured in 2i (Figure 5A). We note that *Uhrf1* transcript levels in 2i were similar for both WT and 3B3L mESCs more consistent with the Prdm14 repression model of hypomethylation in 2i, rather than a model involving downregulation of Uhrf1 (von Meyenn et al., 2016).

Consistent with the model where DNA methylation focuses polycomb targeting, ChIP-sequencing revealed that the maintenance of serum-level DNA methylation levels under 2i conditions in 3B3L cells also resulted in the observed H3K27me3 deposition being largely retained at polycomb target loci (Figures 5B and 5C). The H3K27me3 profiles at CGI that we observed in our rescued 3B3L cells parallel those observed when *de novo* Dnmt activities were reintroduced in *Dnmt3a*$^{-/-}$, *Dnmt3b*$^{-/-}$ hypomethylated mESCs (King et al., 2016).

Consistent with the role of polycomb in mediating chromatin compaction, FISH revealed that the *HoxD* locus is retained in a compact chromatin conformation when 3B3L cells are grown in 2i, contrasting with the decompaction seen at this locus when WT ESCs are grown in these culture conditions (Figure 5D). Inter-probe distances measured across *HoxD* were not significantly different between 3B3L cells grown in serum or 2i (Figures 5E and S5A). This result was also confirmed utilizing a cell line in which *Dnmt3a* and *Dnmt3l* transgenes were exogenously expressed from a constitutive promoter (Figure S5B).

Similarly, in contrast to the loss of long-range clustering between distant polycomb sites such as *En2*, *Shh*, and *Mnx1* seen in WT ESCs in 2i (Figure 4B), inter-probe distances were not increased when 3B3L cells were cultured in 2i (Figure 5F) and the clustering of all three loci together was maintained (Figure 5G). This is consistent with the maintenance of H3K27me3 at these regions in 3B3L cells cultured under 2i conditions (Figure S5C).

The Phenotype of 2i ESCs Is Driven by Culture Conditions, Not the Epigenome or 3D Chromatin Organization

Using 3B3L cells, we are able to grow mESCs in 2i culture conditions and largely maintain the epigenome and 3D genome organization of ESCs grown in serum. To determine whether the phenotype of these cells is determined by the epigenome and 3D genome organization or by the 2i condition and its impact on signaling, we first analyzed features characteristic of the 2i naive ground state of pluripotency.

3B3L cells still appear to exhibit hallmarks of the 2i ground state including upregulation of *Prdm14* (Figure 6A) and characteristic spheroid colony morphology. There was also uniform staining for ESRRB in 3B3L cells growing in 2i, contrasting the heterogeneous staining seen in serum-grown cells (Figure 6B). Serum and 2i mESCs have distinct transcriptional profiles (Marks et al., 2012). To determine whether the transcriptional profile of 3B3L cells in 2i more closely resembles that of mESCs with a similar epigenome and 3D organization (serum ESCs), or that of mESCs grown under similar signaling blockade (2i), we compared RNA sequencing (RNA-seq) data obtained from 3B3L and WT mESCs in 2i conditions. Principal-component analysis showed that the 3B3L/2i transcriptome clusters with that of WT (J1) cells in the same condition, rather than with that of 3B3L cells grown in serum (Figure 6C). These results imply that the serum-like epigenome and 3D genome organization of 3B3L cells growing in 2i conditions has little or no effect on the naive pluripotency transcriptional state of these cells.

DISCUSSION

The observed patterns of DNA/histone modification profiled across the genome, and spatial genome organization assayed by imaging or chromosome conformation capture assays, often correlate with patterns of gene regulation. However, experiments that determine whether there is a causal relationship between the epigenome, 3D genome, and gene regulation are often lacking.

DNA Methylation Impacts on 3D Genome Organization via Polycomb

As previously established by us and others (Brinkman et al., 2012; Jermann et al., 2014; Marks et al., 2012; Reddington et al., 2013, 2014), DNA methylation has a profound effect on the distribution of polycomb (H3K27 tri-methylation) across the mammalian genome, including in ESCs. This is likely to be as a result of both the specific targeting of PRC2 and PRC1 to CGIs (Farcas et al., 2012; Riising et al., 2014) and the generalized affinity of polycomb complexes for chromatin (Blackledge et al., 2015).

Here, we have shown, both by imaging at a few specific exemplar loci and genome wide using Hi-C, that the altered epigenome of 2i ESCs influences the 3D organization of the genome; specifically, a loss of both local chromatin compaction at polycomb target loci (Figures 1 and 3) and long-range polycomb-mediated chromatin contacts (Figure 4). We also show that the loss of chromatin compaction at polycomb target loci, such as *Hox* loci, in naive pluripotency reflects their chromatin conformation *in vivo* in hypomethylated preimplantation blastocysts (Figure 2).

In contrast to polycomb target loci, chromatin compaction at a control non-polycomb target locus was not significantly different between mESCs grown in the serum versus 2i (Figure 1). This suggests that chromatin decompaction is not a result of a global alteration in the 3D chromatin organization of naive 2i cells. It also demonstrates that the significant loss of DNA methylation across a genomic region has no detectable effect

OPEN
ACCESS
CellPress

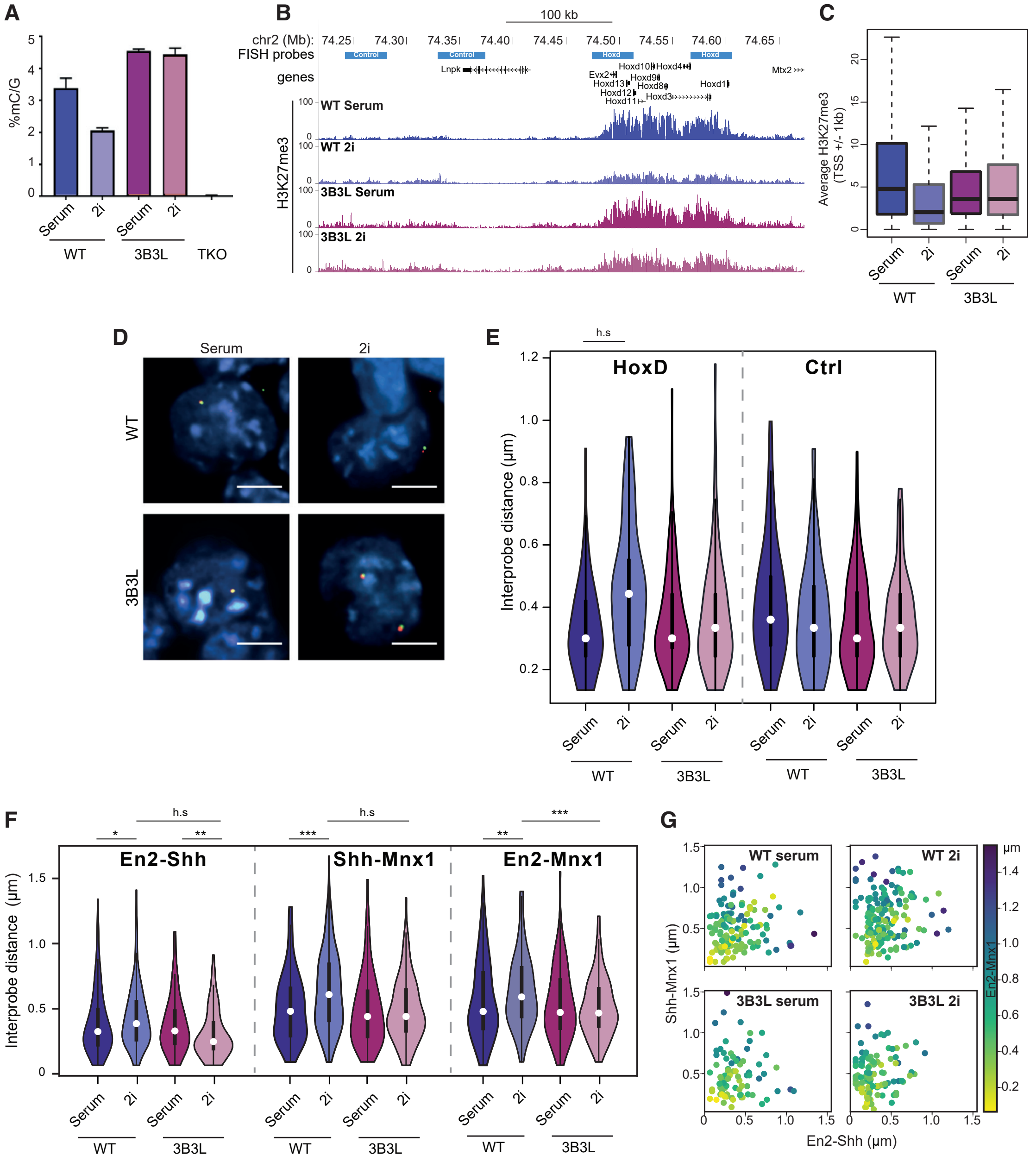

Figure 5. Maintenance of the Epigenetic and 3D Landscape in 2i

(A) DNA methylation measured by mass spectrometry showing global levels of methylated cytosine in WT and 3B3L cells under serum/LIF and 2i/LIF conditions (as well as negative control TKOs, which lack all the active DNMTs; Tsumura et al., 2006). Values represent the percentage of methylated cytosine normalized to total guanine. The mean of two technical replicates is shown, with error bars representing the standard deviation of the mean.

(B) UCSC genome browser screen shot at the *HoxD* locus showing H3K27me3 ChIP-seq in WT and 3B3L cells under serum/LIF and 2i/LIF conditions. Data for wild-type (WT) cells are from Marks et al. (2012). Data are binned into 200-bp windows and normalized by total read count with reads from matching input samples subtracted.

(C) Boxplots representing average H3K27me3 signal on promoters (+/− 1 kb from transcription start site [TSS]) for all promoters in WT or 3B3L cells under serum or 2i conditions.

(D) Representative images of nuclei after FISH with probes for *HoxD* from WT and 3B3L cells grown in serum or 2i. Scale bars represent 10 μm.

(E) Violin plots showing distribution of inter-probe distances at the *HoxD* and a control (Ctrl) locus for WT J1 and 3B3L cells cultured in serum/LIF and 2i/LIF. h.s. = $p < 0.0001$. Biological replicate for 3B3L cells, and data for 3A3L cells are in Figure S5.

(F) Same as in (E), but for probes to *En2*, *Shh*, and *Mnx1*; * $p<0.05$, ** $p<0.01$, *** $p<0.001$ and h.s. $p<0.0001$.

(G) Scatterplots showing individual measurements for data in (F), with two distances shown along the axes and one (*En2-Mnx1*) color coded.

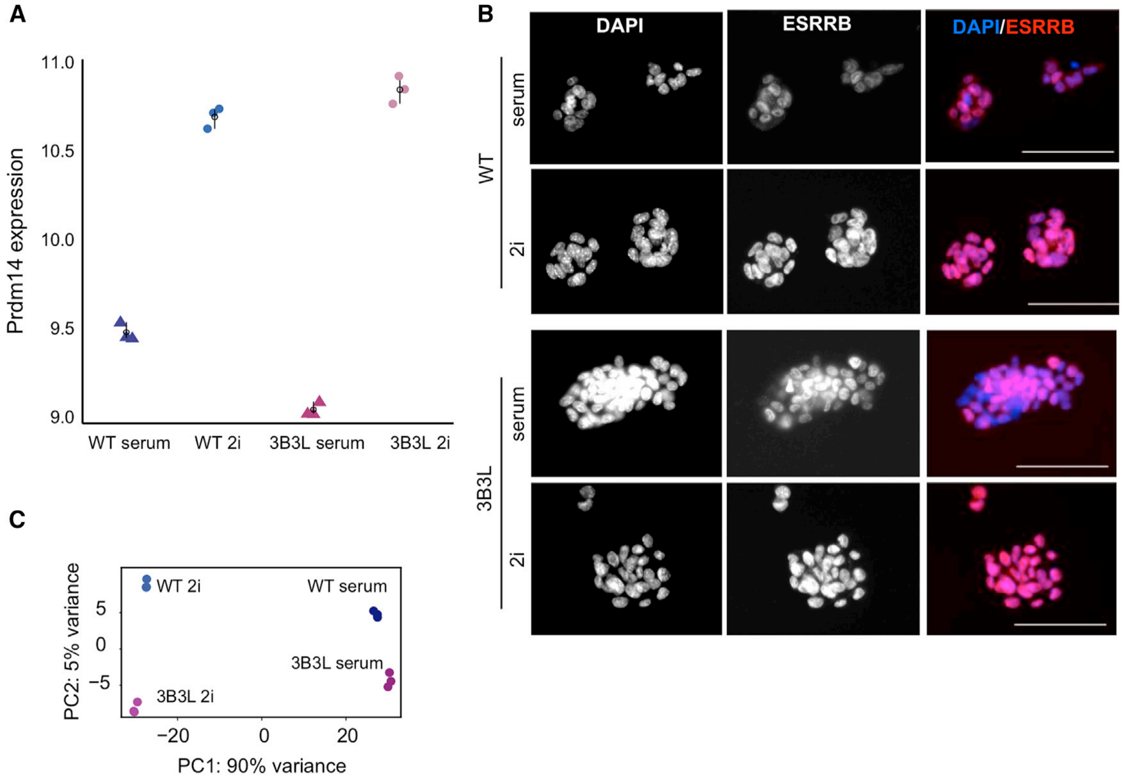

Figure 6. Analysis of the Functional State of 3B3L mESCs in 2i

(A) Regularized log (rlog) transformed expression value for Prdm14 in WT and 3B3L cells cultured in serum or 2i. Error bars show mean and bootstrapped 95% confidence intervals for each cell type and treatment group. Data are from three biological replicates

(B) ESRRB staining in 3B3L cells under 2i or serum conditions. Nuclei are counterstained with DAPI. Exposure times for the TxRed channel (ESRRB) were matched between conditions. Scale bars represent 100 μm.

(C) Principal-component analysis (PCA) of the transcriptome (RNA-seq) of wild-type (WT) J1 and 3B3L mESCs cultured in serum or 2i. Data are from three biological replicates.

on chromatin compaction, as assayed at a cytological level. This is consistent with the finding that chromatin compaction, as assayed by nuclease sensitivity and sucrose gradient sedimentation, in mammalian cells is also not affected by the loss of DNA methylation (Gilbert et al., 2007).

The Epigenome and 3D Genome Do Not Affect the Naive Pluripotency Functional State

By manipulating the epigenome (DNA methylation and H3K27me3 distribution) of mESCs grown in 2i conditions, we have been able to demonstrate that changes in 3D genome organization that occur as ESCs transition between primed and naive pluripotency are a downstream consequence of the shifting epigenome. Constitutive expression of *de novo* DNA methyltransferases during the conversion to 2i conditions largely prevents the changes to DNA methylation and polycomb targeting normally seen for WT mESCs in these culture conditions (Figure 5). This is then reflected in 3D genome organization; in 3B3L mESCs cultured in 2i, *Hox* loci retain their local chromatin compaction and long-range clustering of polycomb sites is preserved (Figure 5).

However, this "serum-like" epigenome and 3D genome organization that we have imposed on ESCs growing in 2i does not detectably affect the transcriptional state of the ESCs. They maintain their high and homogeneous expression of pluripotency markers and the transcriptome of these cells resembles that of the ground state (2i), not that of primed (serum) ESCs (Figure 6). This is consistent with the observation that the decrease of H3K27me3 at gene promoters is not generally associated with transcription activation of these loci under 2i conditions (Galonska et al., 2015; Marks et al., 2012; van Mierlo et al., 2019). We presume that the transcriptional network driven by the defined 2i signaling environment predominates over any instructive information in the epigenome or 3D genome.

Our data demonstrate that 3D genome organization is an emergent property of the epigenome and that the consequences of perturbing one part of the epigenome (DNA methylation) cannot be considered in isolation. Rather, the impact of one epigenetic system on other epigenetic systems (e.g., polycomb) and the related changes in 3D genome organization must be considered together. Our findings also caution against over-interpreting the functional significance of the epigenome and 3D genome organization—at least in ESCs. It will now be interesting to establish how DNA methylation and polycomb become increasingly functionally

important for gene regulation as development progresses (Greenberg et al., 2017).

STAR★METHODS

Detailed methods are provided in the online version of this paper and include the following:

- KEY RESOURCES TABLE
- LEAD CONTACT AND MATERIALS AVAILABILITY
- MATERIALS AVAILABILITY
- EXPERIMENTAL MODEL AND SUBJECT DETAILS
  - Animals
  - Cell Lines
- METHODS DETAILS
  - FISH
  - Image Capture
  - Immunocytochemistry
  - H3K27me3 ChIP-Seq
  - RNA-Seq
  - DNA Methylation by Mass Spectrometry
  - *In Situ* Hi-C
- QUANTIFICATION AND STATISTICAL ANALYSIS
  - FISH Image Analysis
  - Hi-C Data Analysis
  - RNA-Seq Analysis
- DATA AND CODE AVAILABILITY

SUPPLEMENTAL INFORMATION

Supplemental Information can be found online at https://doi.org/10.1016/j.celrep.2019.10.031.

ACKNOWLEDGMENTS

We are grateful to Nezar Abdennur, Anton Goloborodko, and Maxim Imakaev for advice on Hi-C data analysis, and to Sergey Venev for prompt help with loop annotation. We are grateful to Johanna Gassler for advice with FISH on mouse embryos. We thank other W.A.B. and R.R.M. lab members for discussions. K.M. was funded by a PhD studentship from the UK Medical Research Council (MRC). I.M.F. was funded by a PhD studentship from the Darwin Trust. Work in S.P.'s lab is supported by the BBSRC, and BHF. Work in the R.R.M. lab is funded by an MRC University Unit grant (MC_PC_U127574433 and MC_UU_00007/17). I.R.A. is funded by MRC University Unit grants (MC_PC_U127580973 and MC_UU_00007/6). Work in the W.A.B. lab is funded by an MRC University Unit grant (MC_UU_00007/2).

AUTHOR CONTRIBUTIONS

The study was conceived, designed, and supervised together by R.R.M. and W.A.B. FISH analysis of *Hox* loci was performed and analyzed by K.M. FISH analysis of *En2*, *Shh*, and *Mnx1* was performed by I.W. Hi-C was performed by I.M.F. and K.M. and analyzed by I.M.F. H.K.M., R.S., and S.P. created and characterized stable cell lines 3A3L and 3B3L, prepared samples for RNA-seq, and assisted with experimental design and protocols. I.R.A. provided blastocysts for FISH. K.M., I.M.F., G.R.G., and W.A.B. prepared figures. RNA-seq analysis was performed by G.R.G. J.P.T. and R.S.I. performed computational genomic analysis. K.M., R.R.M., W.A.B., and I.M.F. wrote the manuscript with input from all authors.

DECLARATION OF INTERESTS

The authors declare no competing interests.

Received: January 28, 2019
Revised: September 9, 2019
Accepted: October 9, 2019
Published: November 12, 2019

REFERENCES

Abdennur, N., and Mirny, L. (2019). Cooler: scalable storage for Hi-C data and other genomically-labeled arrays. Bioinformatics, Published online July 10, 2019. https://doi.org/10.1093/bioinformatics/btz540.

Atlasi, Y., Megchelenbrink, W., Peng, T., Habibi, E., Joshi, O., Wang, S.Y., Wang, C., Logie, C., Poser, I., Marks, H., and Stunnenberg, H.G. (2019). Epigenetic modulation of a hardwired 3D chromatin landscape in two naive states of pluripotency. Nat. Cell Biol. *21*, 568–578.

Blackledge, N.P., Rose, N.R., and Klose, R.J. (2015). Targeting Polycomb systems to regulate gene expression: modifications to a complex story. Nat. Rev. Mol. Cell Biol. *16*, 643–649.

Boettiger, A.N., Bintu, B., Moffitt, J.R., Wang, S., Beliveau, B.J., Fudenberg, G., Imakaev, M., Mirny, L.A., Wu, C.T., and Zhuang, X. (2016). Super-resolution imaging reveals distinct chromatin folding for different epigenetic states. Nature *529*, 418–422.

Bonev, B., Mendelson Cohen, N., Szabo, Q., Fritsch, L., Papadopoulos, G.L., Lubling, Y., Xu, X., Lv, X., Hugnot, J.P., Tanay, A., and Cavalli, G. (2017). Multiscale 3D Genome Rewiring during Mouse Neural Development. Cell *171*, 557–572.e24.

Brinkman, A.B., Gu, H., Bartels, S.J., Zhang, Y., Matarese, F., Simmer, F., Marks, H., Bock, C., Gnirke, A., Meissner, A., and Stunnenberg, H.G. (2012). Sequential ChIP-bisulfite sequencing enables direct genome-scale investigation of chromatin and DNA methylation cross-talk. Genome Res. *22*, 1128–1138.

Canham, M.A., Sharov, A.A., Ko, M.S., and Brickman, J.M. (2010). Functional heterogeneity of embryonic stem cells revealed through translational amplification of an early endodermal transcript. PLoS Biol. *8*, e1000379.

Cohen, I., Zhao, D., Bar, C., Valdes, V.J., Dauber-Decker, K.L., Nguyen, M.B., Nakayama, M., Rendl, M., Bickmore, W.A., Koseki, H., et al. (2018). PRC1 Fine-tunes Gene Repression and Activation to Safeguard Skin Development and Stem Cell Specification. Cell Stem Cell *22*, 726–739.e7.

Denholtz, M., Bonora, G., Chronis, C., Splinter, E., de Laat, W., Ernst, J., Pellegrini, M., and Plath, K. (2013). Long-range chromatin contacts in embryonic stem cells reveal a role for pluripotency factors and polycomb proteins in genome organization. Cell Stem Cell *13*, 602–616.

Du, Z., Zheng, H., Huang, B., Ma, R., Wu, J., Zhang, X., He, J., Xiang, Y., Wang, Q., Li, Y., et al. (2017). Allelic reprogramming of 3D chromatin architecture during early mammalian development. Nature *547*, 232–235.

Eskeland, R., Leeb, M., Grimes, G.R., Kress, C., Boyle, S., Sproul, D., Gilbert, N., Fan, Y., Skoultchi, A.I., Wutz, A., and Bickmore, W.A. (2010). Ring1B compacts chromatin structure and represses gene expression independent of histone ubiquitination. Mol. Cell *38*, 452–464.

Farcas, A.M., Blackledge, N.P., Sudbery, I., Long, H.K., McGouran, J.F., Rose, N.R., Lee, S., Sims, D., Cerase, A., Sheahan, T.W., et al. (2012). KDM2B links the Polycomb Repressive Complex 1 (PRC1) to recognition of CpG islands. eLife *1*, e00205.

Ficz, G., Hore, T.A., Santos, F., Lee, H.J., Dean, W., Arand, J., Krueger, F., Oxley, D., Paul, Y.L., Walter, J., et al. (2013). FGF signaling inhibition in ESCs drives rapid genome-wide demethylation to the epigenetic ground state of pluripotency. Cell Stem Cell *13*, 351–359.

Flyamer, I.M., Gassler, J., Imakaev, M., Brandão, H.B., Ulianov, S.V., Abdennur, N., Razin, S.V., Mirny, L.A., and Tachibana-Konwalski, K. (2017). Single-nucleus Hi-C reveals unique chromatin reorganization at oocyte-to-zygote transition. Nature *544*, 110–114.

Flyamer, I.M., Robert, S., Illingworth, R.S., Wendy, A., and Bickmore, W.A. (2019). *Coolpup.py* – a versatile tool to perform pile-up analysis of Hi-C data. bioRxiv. https://doi.org/10.1101/586537.

Francis, N.J., Kingston, R.E., and Woodcock, C.L. (2004). Chromatin compaction by a polycomb group protein complex. Science *306*, 1574–1577.

Galonska, C., Ziller, M.J., Karnik, R., and Meissner, A. (2015). Ground State Conditions Induce Rapid Reorganization of Core Pluripotency Factor Binding before Global Epigenetic Reprogramming. Cell Stem Cell *17*, 462–470.

Gilbert, N., Thomson, I., Boyle, S., Allan, J., Ramsahoye, B., and Bickmore, W.A. (2007). DNA methylation affects nuclear organization, histone modifications, and linker histone binding but not chromatin compaction. J. Cell Biol. *177*, 401–411.

Grabole, N., Tischler, J., Hackett, J.A., Kim, S., Tang, F., Leitch, H.G., Magnúsdóttir, E., and Surani, M.A. (2013). Prdm14 promotes germline fate and naive pluripotency by repressing FGF signalling and DNA methylation. EMBO Rep. *14*, 629–637.

Graf, U., Casanova, E.A., Wyck, S., Dalcher, D., Gatti, M., Vollenweider, E., Okoniewski, M.J., Weber, F.A., Patel, S.S., Schmid, M.W., et al. (2017). Pramel7 mediates ground-state pluripotency through proteasomal-epigenetic combined pathways. Nat. Cell Biol. *19*, 763–773.

Greenberg, M.V., Glaser, J., Borsos, M., Marjou, F.E., Walter, M., Teissandier, A., and Bourc'his, D. (2017). Transient transcription in the early embryo sets an epigenetic state that programs postnatal growth. Nat. Genet. *49*, 110–118.

Habibi, E., Brinkman, A.B., Arand, J., Kroeze, L.I., Kerstens, H.H., Matarese, F., Lepikhov, K., Gut, M., Brun-Heath, I., Hubner, N.C., et al. (2013). Whole-genome bisulfite sequencing of two distinct interconvertible DNA methylomes of mouse embryonic stem cells. Cell Stem Cell *13*, 360–369.

Hackett, J.A., and Surani, M.A. (2014). Regulatory principles of pluripotency: from the ground state up. Cell Stem Cell *15*, 416–430.

Hayashi, K., de Sousa Lopes, S.M.C., Tang, F., Lao, K., and Surani, M.A. (2008). Dynamic equilibrium and heterogeneity of mouse pluripotent stem cells with distinct functional and epigenetic states. Cell Stem Cell *3*, 391–401.

Illingworth, R.S., Botting, C.H., Grimes, G.R., Bickmore, W.A., and Eskeland, R. (2012). PRC1 and PRC2 are not required for targeting of H2A.Z to developmental genes in embryonic stem cells. PLoS ONE *7*, e34848.

Illingworth, R.S., Moffat, M., Mann, A.R., Read, D., Hunter, C.J., Pradeepa, M.M., Adams, I.R., and Bickmore, W.A. (2015). The E3 ubiquitin ligase activity of RING1B is not essential for early mouse development. Genes Dev. *29*, 1897–1902.

Jackson, M., Krassowska, A., Gilbert, N., Chevassut, T., Forrester, L., Ansell, J., and Ramsahoye, B. (2004). Severe global DNA hypomethylation blocks differentiation and induces histone hyperacetylation in embryonic stem cells. Mol. Cell. Biol. *24*, 8862–8871.

Jermann, P., Hoerner, L., Burger, L., and Schübeler, D. (2014). Short sequences can efficiently recruit histone H3 lysine 27 trimethylation in the absence of enhancer activity and DNA methylation. Proc. Natl. Acad. Sci. USA *111*, E3415–E3421.

Joshi, O., Wang, S.Y., Kuznetsova, T., Atlasi, Y., Peng, T., Fabre, P.J., Habibi, E., Shaik, J., Saeed, S., Handoko, L., et al. (2015). Dynamic Reorganization of Extremely Long-Range Promoter-Promoter Interactions between Two States of Pluripotency. Cell Stem Cell *17*, 748–757.

Ke, Y., Xu, Y., Chen, X., Feng, S., Liu, Z., Sun, Y., Yao, X., Li, F., Zhu, W., Gao, L., et al. (2017). 3D Chromatin Structures of Mature Gametes and Structural Reprogramming during Mammalian Embryogenesis. Cell *170*, 367–381.e20.

King, A.D., Huang, K., Rubbi, L., Liu, S., Wang, C.Y., Wang, Y., Pellegrini, M., and Fan, G. (2016). Reversible Regulation of Promoter and Enhancer Histone Landscape by DNA Methylation in Mouse Embryonic Stem Cells. Cell Rep. *17*, 289–302.

Kundu, S., Ji, F., Sunwoo, H., Jain, G., Lee, J.T., Sadreyev, R.I., Dekker, J., and Kingston, R.E. (2017). Polycomb Repressive Complex 1 Generates Discrete Compacted Domains that Change during Differentiation. Mol. Cell *65*, 432–446.e5.

Leeb, M., and Wutz, A. (2007). Ring1B is crucial for the regulation of developmental control genes and PRC1 proteins but not X inactivation in embryonic cells. J Cell Biol. *178*, 219–229.

Leitch, H.G., McEwen, K.R., Turp, A., Encheva, V., Carroll, T., Grabole, N., Mansfield, W., Nashun, B., Knezovich, J.G., Smith, A., et al. (2013). Naive pluripotency is associated with global DNA hypomethylation. Nat. Struct. Mol. Biol. *20*, 311–316.

Li, H., Liefke, R., Jiang, J., Kurland, J.V., Tian, W., Deng, P., Zhang, W., He, Q., Patel, D.J., Bulyk, M.L., et al. (2017). Polycomb-like proteins link the PRC2 complex to CpG islands. Nature *549*, 287–291.

Lieberman-Aiden, E., van Berkum, N.L., Williams, L., Imakaev, M., Ragoczy, T., Telling, A., Amit, I., Lajoie, B.R., Sabo, P.J., Dorschner, M.O., et al. (2009). Comprehensive mapping of long-range interactions reveals folding principles of the human genome. Science *326*, 289–293.

Marks, H., Kalkan, T., Menafra, R., Denissov, S., Jones, K., Hofemeister, H., Nichols, J., Kranz, A., Stewart, A.F., Smith, A., and Stunnenberg, H.G. (2012). The transcriptional and epigenomic foundations of ground state pluripotency. Cell *149*, 590–604.

Messerschmidt, D.M., Knowles, B.B., and Solter, D. (2014). DNA methylation dynamics during epigenetic reprogramming in the germline and preimplantation embryos. Genes Dev. *28*, 812–828.

Morey, C., Da Silva, N.R., Perry, P., and Bickmore, W.A. (2007). Nuclear reorganisation and chromatin decondensation are conserved, but distinct, mechanisms linked to Hox gene activation. Development *134*, 909–919.

Morgani, S.M., Canham, M.A., Nichols, J., Sharov, A.A., Migueles, R.P., Ko, M.S., and Brickman, J.M. (2013). Totipotent embryonic stem cells arise in ground-state culture conditions. Cell Rep. *3*, 1945–1957.

Noordermeer, D., Leleu, M., Splinter, E., Rougemont, J., De Laat, W., and Duboule, D. (2011). The dynamic architecture of Hox gene clusters. Science *334*, 222–225.

Okano, M., Bell, D.W., Haber, D.A., and Li, E. (1999). DNA methyltransferases Dnmt3a and Dnmt3b are essential for de novo methylation and mammalian development. Cell *99*, 247–257.

Probst, A.V., Santos, F., Reik, W., Almouzni, G., and Dean, W. (2007). Structural differences in centromeric heterochromatin are spatially reconciled on fertilisation in the mouse zygote. Chromosoma *116*, 403–415.

Rao, S.S., Huntley, M.H., Durand, N.C., Stamenova, E.K., Bochkov, I.D., Robinson, J.T., Sanborn, A.L., Machol, I., Omer, A.D., Lander, E.S., and Aiden, E.L. (2014). A 3D map of the human genome at kilobase resolution reveals principles of chromatin looping. Cell *159*, 1665–1680.

Reddington, J.P., Perricone, S.M., Nestor, C.E., Reichmann, J., Youngson, N.A., Suzuki, M., Reinhardt, D., Dunican, D.S., Prendergast, J.G., Mjoseng, H., et al. (2013). Redistribution of H3K27me3 upon DNA hypomethylation results in de-repression of Polycomb target genes. Genome Biol. *14*, R25.

Reddington, J.P., Sproul, D., and Meehan, R.R. (2014). DNA methylation reprogramming in cancer: does it act by re-configuring the binding landscape of Polycomb repressive complexes? BioEssays *36*, 134–140.

Riising, E.M., Comet, I., Leblanc, B., Wu, X., Johansen, J.V., and Helin, K. (2014). Gene silencing triggers polycomb repressive complex 2 recruitment to CpG islands genome wide. Mol. Cell *55*, 347–360.

Rossant, J., Sanford, J.P., Chapman, V.M., and Andrews, G.K. (1986). Undermethylation of structural gene sequences in extraembryonic lineages of the mouse. Dev. Biol. *117*, 567–573.

Schoeftner, S., Sengupta, A.K., Kubicek, S., Mechtler, K., Spahn, L., Koseki, H., Jenuwein, T., and Wutz, A. (2006). Recruitment of PRC1 function at the initiation of X inactivation independent of PRC2 and silencing. EMBO J. *25*, 3110–3122.

Schoenfelder, S., Sugar, R., Dimond, A., Javierre, B.M., Armstrong, H., Mifsud, B., Dimitrova, E., Matheson, L., Tavares-Cadete, F., Furlan-Magaril, M., et al. (2015). Polycomb repressive complex PRC1 spatially constrains the mouse embryonic stem cell genome. Nat. Genet. *47*, 1179–1186.

Schuettengruber, B., Bourbon, H.M., Di Croce, L., and Cavalli, G. (2017). Genome Regulation by Polycomb and Trithorax: 70 Years and Counting. Cell *171*, 34–57.

Thomson, J.P., Fawkes, A., Ottaviano, R., Hunter, J.M., Shukla, R., Mjoseng, H.K., Clark, R., Coutts, A., Murphy, L., and Meehan, R.R. (2015). DNA

immunoprecipitation semiconductor sequencing (DIP-SC-seq) as a rapid method to generate genome wide epigenetic signatures. Sci. Rep. *5*, 9778.

Tsumura, A., Hayakawa, T., Kumaki, Y., Takebayashi, S., Sakaue, M., Matsuoka, C., Shimotohno, K., Ishikawa, F., Li, E., Ueda, H.R., et al. (2006). Maintenance of self-renewal ability of mouse embryonic stem cells in the absence of DNA methyltransferases Dnmt1, Dnmt3a and Dnmt3b. Genes Cells *11*, 805–814.

van Mierlo, G., Dirks, R.A.M., De Clerck, L., Brinkman, A.B., Huth, M., Kloet, S.L., Saksouk, N., Kroeze, L.I., Willems, S., Farlik, M., et al. (2019). Integrative Proteomic Profiling Reveals PRC2-Dependent Epigenetic Crosstalk Maintains Ground-State Pluripotency. Cell Stem Cell *24*, 123–137.e8.

Vieux-Rochas, M., Fabre, P.J., Leleu, M., Duboule, D., and Noordermeer, D. (2015). Clustering of mammalian Hox genes with other H3K27me3 targets within an active nuclear domain. Proc. Natl. Acad. Sci. USA *112*, 4672–4677.

von Meyenn, F., Iurlaro, M., Habibi, E., Liu, N.Q., Salehzadeh-Yazdi, A., Santos, F., Petrini, E., Milagre, I., Yu, M., Xie, Z., et al. (2016). Impairment of DNA Methylation Maintenance Is the Main Cause of Global Demethylation in Naive Embryonic Stem Cells. Mol. Cell *62*, 848–861.

Williamson, I., Eskeland, R., Lettice, L.A., Hill, A.E., Boyle, S., Grimes, G.R., Hill, R.E., and Bickmore, W.A. (2012). Anterior-posterior differences in HoxD chromatin topology in limb development. Development *139*, 3157–3167.

Williamson, I., Berlivet, S., Eskeland, R., Boyle, S., Illingworth, R.S., Paquette, D., Dostie, J., and Bickmore, W.A. (2014). Spatial genome organization: contrasting views from chromosome conformation capture and fluorescence in situ hybridization. Genes Dev. *28*, 2778–2791.

Wills, J., Edwards-Hicks, J., and Finch, A.J. (2017). AssayR: A Simple Mass Spectrometry Software Tool for Targeted Metabolic and Stable Isotope Tracer Analyses. Anal. Chem. *89*, 9616–9619.

Wongtawan, T., Taylor, J.E., Lawson, K.A., Wilmut, I., and Pennings, S. (2011). Histone H4K20me3 and HP1α are late heterochromatin markers in development, but present in undifferentiated embryonic stem cells. J. Cell Sci. *124*, 1878–1890.

Wray, J., Kalkan, T., Gomez-Lopez, S., Eckardt, D., Cook, A., Kemler, R., and Smith, A. (2011). Inhibition of glycogen synthase kinase-3 alleviates Tcf3 repression of the pluripotency network and increases embryonic stem cell resistance to differentiation. Nat. Cell Biol. *13*, 838–845.

Wutz, A., and Jaenisch, R. (2000). A shift from reversible to irreversible X inactivation is triggered during ES cell differentiation. Mol Cell. *5*, 695–705.

Yamaji, M., Ueda, J., Hayashi, K., Ohta, H., Yabuta, Y., Kurimoto, K., Nakato, R., Yamada, Y., Shirahige, K., and Saitou, M. (2013). PRDM14 ensures naive pluripotency through dual regulation of signaling and epigenetic pathways in mouse embryonic stem cells. Cell Stem Cell *12*, 368–382.

Ying, Q.L., and Smith, A. (2017). The Art of Capturing Pluripotency: Creating the Right Culture. Stem Cell Reports *8*, 1457–1464.

Ying, Q.L., Wray, J., Nichols, J., Batlle-Morera, L., Doble, B., Woodgett, J., Cohen, P., and Smith, A. (2008). The ground state of embryonic stem cell self-renewal. Nature *453*, 519–523.

Zhang, Y., Xiang, Y., Yin, Q., Du, Z., Peng, X., Wang, Q., Fidalgo, M., Xia, W., Li, Y., Zhao, Z.A., et al. (2018). Dynamic epigenomic landscapes during early lineage specification in mouse embryos. Nat. Genet. *50*, 96–105.

STAR★METHODS

KEY RESOURCES TABLE

REAGENT or RESOURCE	SOURCE	IDENTIFIER
Antibodies		
ESRRB (Human ERRβ, clone H6705)	Perscus proteomics, mouse,	PP-H6705; RRID: AB_2100412
RING1B (Rnf2)	MBL	D139-3; RRID: AB_592650
H3K27me3	Millipore	07-449; RRID: AB_310624
Donkey anti-mouse Alexafluor 555	Thermo Fisher	A31570; RRID: AB_2536180
Bacterial and Virus Strains		
Fosmid FISH probes	BACPAC resource	See Table S3
Chemicals, Peptides, and Recombinant Proteins		
DMEM, high glucose, pyruvate	GIBCO	41966029
Glasgow's MEM	GIBCO	21710025
DMEM/F-12	GIBCO	11320033
Neurobasal Medium	GIBCO	21103049
N-2 Supplement (100X)	GIBCO	17502001
B-27 Supplement (50X), serum free	GIBCO	17504044
Bovine Albumin Fraction V (7.5% solution)	GIBCO	15260037
Fetal calf serum (Myoclone)	Thermo Fisher	10081-073
Stemolecule PD0325901	Stemgent	040006
Stemolecule CHIR99021	Stemgent	040004
1-Thioglycerol	Sigma	M6145
ESGRO® Recombinant Mouse LIF Protein	Millipore	ESG1106
Biotin-16-dUTP	Roche	11093070910
Digoxigenin-11-dUTP	Roche	11573152910
Green 500 dUTP	Enzo Life Sciences	ENZ-42845
ChromaTide Alexa Fluor 594-5-dUTP	Invitrogen, ThermoFisher	C11400
Mouse Cot-1 DNA	Invitrogen, ThermoFisher	18440-016
Protein G Dynabeads	Invitrogen, ThermoFisher	10003D
HPLC grade water	Chromasolv, Sigma	7732-18-5
T7 DNA Polymerase	Thermo Scientific	EP0081
Agencourt AMPure XP beads	Beckman Coulter	10136224
Ion Xpress Barcode adaptors	Thermo Fisher	4471250
Formaldehyde	CALBIOCHEM	344198
IGEPAL CA-630	Sigma	I8896
Halt Protease Inhibitor Cocktail	Thermo Scientific	78430
NEBuffer 3	New England Biolabs	B7003S
Triton X-100	Sigma	93443
DpnII with buffer	New England Biolabs	R0543M
dNTPs	Life Technologies	0297018
Biotin-14-dATP	Invitrogen	*19524016*
DNA Polymerase I Klenow Fragment	New England Biolabs	*M0210L*
T4 DNA Ligase Buffer	New England Biolabs	B0202S
T4 DNA Ligase	New England Biolabs	*M0202M*
Dynabeads MyOne Streptavidin T1	Life Technologies	*65602*
T4 Polynucleotide Kinase	New England Biolabs	*M0201L*

(*Continued on next page*)

Continued		
REAGENT or RESOURCE	SOURCE	IDENTIFIER
T4 DNA Polymerase	New England Biolabs	*M0203L*
NEBuffer 2	New England Biolabs	B7002S
DNA polymerase I Klenow (3'→5' exo-) fragment	New England Biolabs	*M0212L*
Quick Ligation Kit	New England Biolabs	*M2200L*
Q5® High-Fidelity DNA Polymerase	New England Biolabs	*M0491L*
Critical Commercial Assays		
TURBO DNA-free Kit	Ambion	AM1907
TruSeq Stranded mRNA Library Kit	Illumina	20020594
RNeasy kit	QIAGEN	74106
MinElute PCR Purification Kit	QIAGEN	28004
Qubit dsDNA HS Assay Kit	Invitrogen	Q32854
Qubit RNA HS Assay Kit	Invitrogen	Q32852
Ion XpressPlus fragment library kit	Thermo Fisher	4471269
Amicon Filter Units 30K 500 μl	Millipore	UFC5030BK
Deposited Data		
Hi-C E14 mESCs grown in serum and 2i media	https://www.ncbi.nlm.nih.gov/geo	GSE124342
RNA-Seq	https://www.ncbi.nlm.nih.gov/geo	GSE121171
H3K27me3 ChIP-seq in serum and 2i media	https://www.ncbi.nlm.nih.gov/geo	GSE72555
Ring1B ChIP-seq	https://www.ncbi.nlm.nih.gov/geo	GSE69978
Experimental Models: Cell Lines		
E14 mESCs	Fiona Kilanowski, IGMM	N/A
WT J1 (clone 36) mESCs	Wutz and Jaenisch, 2000	N/A
Ring1B$^{-/-}$ mESCs	Leeb and Wutz, 2007	N/A
Eed$^{-/-}$ mESCs	Schoeftner et al., 2006	N/A
3B3L cells	Okano et al., 1999; Jackson et al., 2004; and this paper	N/A
Experimental Models: Organisms/Strains		
C57BL/6 mice	Charles River	N/A
Software and Algorithms		
Volocity® 3D Image Analysis Software	PerkinElmer Inc	www.quorumtechnologies.com
Torrent TMP	Github	https://github.com/iontorrent/TMAP
Distiller	Github	https://github.com/mirnylab/distiller-nf
Pairtools	Github	https://github.com/mirnylab/pairtools
Cooler	Github	https://github.com/mirnylab/cooler
Cooltools	Github	https://github.com/mirnylab/cooltools
Coolpup.py	Github	https://github.com/Phlya/coolpuppy
Sailfish	Github	https://github.com/kingsfordgroup/sailfish
Other		
Hypercarb HPLC Column	Thermo Scientific	35003-031030
DNA LoBind tubes	Eppendorf	0030108051

LEAD CONTACT AND MATERIALS AVAILABILITY

Further information and requests for resources and reagents should be directed to, and will be fulfilled, by the Lead Contact, Richard Meehan (Richard.Meehan@igmm.ed.ac.uk).

MATERIALS AVAILABILITY

All unique/stable reagents generated in this study are available from the Lead Contact with a completed Materials Transfer Agreement.

EXPERIMENTAL MODEL AND SUBJECT DETAILS

Animals

C57BL/6 mice were maintained in accordance with institutional guidelines and national regulations. Animal experiments were performed under the authority of UK Home Office project license PPL60/4424 following ethical review by the University of Edinburgh Animal Welfare and Ethical Review Body.

Cell Lines

Male mouse embryonic stem cell lines used in this study are: E14, WT (clone 36), *Ring1B*$^{-/-}$, *Eed*$^{-/-}$ (Eskeland et al., 2010) and WT J1. 3B3L/3A3L cells are DKO (*Dnmt3a*$^{-/-}$, *3b*$^{-/-}$) mESC lines where DNA methylation is maintained with a Dnmt3b/ or Dnmt3a expressing transgene under the control of the CAG promoter (3B3/3A3) (Jackson et al., 2004; Okano et al., 1999) and which were transfected with pCAGGS-*Dnmt3l*-*Flag*-IRES-Blasticidin-polyA, and selected by blasticidin (5 μg/ml) to obtain cell lines with stable expression of *Dnmt3l*.

mESCs were maintained at 37°C with 5% CO2 and passaged every 2-3 days. Serum cells were maintained in either DMEM (in the case of J1-derived lines) or GMEM (in the case of E14-derived lines) (both GIBCO) supplemented with 15% fetal calf serum, 0.1 mM nonessential amino acids (SIGMA), 1 mM sodium Pyruvate (Sigma) 1% Penicillin/Streptomycin, 2 mM L-glutamine, 0.1 mM β-mercaptoethanol (Thermo Fisher), and ESGRO LIF (Millipore) at 1000 U/mL. Cells were either grown on 0.2% gelatin (Sigma) (E14 cells, 3B3L cells) or on mitomyin C-inactivated SNLP feeder cells in the case of serum culture J1/clone36-derived cells. 2i culture conditions include 50% DMEM/F12 (GIBCO), 50% Neurobasal media (GIBCO), 0.5% N2 supplement, 1% B27 & RA (GIBCO), 7.5% BSA (GIBCO), 1% Penicilllin/Streptomycin, 2 mM L-glutamine, 0.15 mM monothioglycerol (Sigma), 1000 U/ml ESGRO LIF (Millipore), 1 μM PD0325901 (MEK inhibitor, Stemgent) and 3 μM CHIR99021 (GSK3 inhibitor, Stemgent). mESCs were passaged every 2-3 days using trypsin/EDTA (Sigma). 2i conversions were carried out for 14 days. To deplete feeder-dependent mESCs of their feeders for analysis/2i-conversion, the culture was plated 3x for 20 mins, in which time the feeders stick to the tissue culture dish and mESCs do not.

METHODS DETAILS

FISH

One million mESCs were plated onto gelatinized slides for 4 h. Cells were fixed in 4% paraformaldehyde (pFA) for 10 min, permeabilized in 0.5% Triton X-100 for 10 min, air-dried and stored at −80°C. Slides were incubated with 100 ug/ml RNaseA in 2 x SSC for 1 h, washed in 2 x SSC and dehydrated through an alcohol series. Slides were then denatured in 70% formamide/2xSSC at 80°C for 30 min. Fosmid clones (Table S3) were prepared and labeled with digoxigenin-11-dUTP or with biotin-16-dUTP as previously described (Morey et al., 2007). Approximately 160 ng of biotin- and digoxigenin-labelled fosmid probes were used per slide, with 16-24 mg of Cot1 DNA (Invitrogen) and 10 mg of salmon sperm DNA. For 4-colour FISH, a similar quantity of the additional fosmid was labelled with either red-dUTP (ChromaTide Alexa FluorTM 594-5-dUTP,Invitrogen) or 5(6)-Carboxyrhodamine Green (Green 500) dUTP (Enzo).

Approximately 150 ng of labeled fosmid probes were used per slide, together with 8 μg of mouse Cot1 DNA (Invitrogen) and 5 μg sonicated salmon sperm DNA. Probes were denatured at 80°C for 5 min, preannealed for 15 min at 37°C and hybridized to the denatured slides overnight (o/n). The following day, the slides were washed in 2x SSC followed by 0.1x SSC and stained in DAPI prior to imaging.

For FISH on blastocysts, an adaptation of previously described protocols was used (Flyamer et al., 2017; Probst et al., 2007). Briefly, 20 female C57BL/6 mice were superovulated and mated with C57BL/6 males, and blastocysts isolated at E3.5 by flushing the uterine horns with FHM media. Blastocysts with visible blastocoels were fixed in 4% pFA and their zona pellucidae removed using Acidic Tyrode’s. The blastocysts were permeabilized in 0.2% Triton X-100 in PBS. Fixed samples were embedded in fibrin clots to attach the blastocysts to slides. Post-fixation was carried out in 2% pFA/ PBS for 30 min. Finally, the slide was rinsed 3x in PBS and stored in PBS at 4°C. FISH was carried out using directly labeled probes described above, with some modifications. The slides were denatured for 45 min.

Image Capture

Images were captured using a Hamamatsu Orca AG CCD camera (Hamamatsu Photonics (UK) Ltd, Welwyn Garden City, UK) and a Zeiss Axioplan II epifluorescence microscope with Plan-neofluar objectives, a 100W Hg source (Carl Zeiss, Welwyn Garden City, UK) and Chroma #83000 triple band pass filter set (Chroma Technology Corp., Rockingham, VT) with the excitation filters installed in a motorized filter wheel (Prior Scientific Instruments, Cambridge, UK). A piezoelectrically driven objective mount (PIFOC model P-721, Physik Instrumente GmbH & Co, Karlsruhe) was used to control movement in the z dimension (with 0.2 μm step).

Immunocytochemistry

mESCs grown on glass coverslips coated with gelatin were fixed with 4% PFA for 20 mins, blocked in 10% donkey serum (Sigma) in 0.1% Triton X-100 for 1 h and incubated o/n with primary antibody detecting ESRRB (Perseus Proteomics, PP-H6705-00) at a 1:500 dilution at 4°C. The following day, samples were incubated with Donkey anti mouse Alexafluor 555 (Cat: A-31570, Thermo Fisher) at room temperature for 1 h. Nuclei were counterstained with DAPI. Imaging was carried out using a Zeiss Axioscope 2 microscope.

H3K27me3 ChIP-Seq

Chromatin prepared from formaldehyde fixed 3B3-3l cells cultured in serum or 2i was fragmented (Covaris sonicator) to a mean fragment size of 200bp. Approximately $5x10^6$ cell equivalents were used for each immunoprecipitation. ChIP was performed using antibody toward H3K27Me3 (Millipore) and Protein G Dynabeads (Thermo Fisher) were used to obtain antibody bound chromatin. Following immunoprecipitation, beads were washed once in X-ChIP wash buffer (150mM NaCl; 10mM Tris pH8; 2mM EDTA; 1% NP40; 0.1% sodium deoxycholate w/v), and once in LiCl wash buffer (100mM Tris pH7.5; 500mM LiCl; 1% NP40; 1% sodium deoxycholate) for 10 min at 4°C each wash. DNA was then reverse crosslinked and eluted from the beads by incubation in elution buffer (1% SDS, 0.1M $NaHCO_3$) followed by treatment with RNase and proteinase K before purification using a QIAGEN minelute kit (QIAGEN) as per manufacturer's instructions and eluting the DNA in 11 μL EB buffer from the kit. Finally, DNA was quantified using a Qubit HS DNA quantification kit (Thermo Fisher) and 1ng DNA was then used to prepare sequencing libraries for Ion Torrent sequencing using the Ion XpressPlus Fragment Library Kit (Thermo Fisher). The DNA was end repaired, purified, and ligated to Ion-compatible barcoded adapters (Ion Xpress Barcode Adapters 1–96: (Thermo Fisher) followed by nick-repair to complete the linkage between adapters and DNA inserts. The adaptor-ligated library was then amplified (10 cycles) and size-selected using two rounds of AMPure XP bead (Beckman Coulter) capture to size-select fragments approximately 100–250bp in length. Samples were pooled at a 1:1 ratio and sequenced on an Ion Proton P1 microwell chip (Thermo Fisher).

Mapping and data normalization were carried out as described previously (Thomson et al., 2015). In short, reads were mapped to the reference genome using the Torrent TMAP software. The data were then binned into 200bp windows across the genome and normalized by total read count. Raw sequencing datasets from published WT E14 mESCs in both serum and 2i were processed in a similar manner (Marks et al., 2012): NCBI GSE23943.

RNA-Seq

RNA was extracted from snap frozen mESC pellets, 3 biological replicates per cell line, using an RNeasy kit (QIAGEN). RNA was quantified by nanodrop and DNA was removed by treatment with Turbo DNA-free reagents (AM1907, Ambion) according to the manufacturer's protocol. Total RNA samples were quantified using the Qubit 2.0 Fluorometer (Thermo Fisher Scientific Inc, Q32866) and the Qubit RNA HS assay kit (Q33855). RNA integrity was assessed using the Agilent 2100 Bioanalyser System (Agilent Technologies Inc, GS2938B) and Agilent RNA 6000 Nano kit (5067-1511).

Sequencing libraries were prepared from 500 ng of each total-RNA sample using the TruSeq Stranded mRNA Library Kit (Illumina Inc, 20020594) according to the provided protocol. Poly-A mRNAs were purified using poly-T oligo attached magnetic beads, and fragmented using divalent cations under elevated temperature and primed with random hexamers. Primed RNA fragments were reverse transcribed into first strand cDNA using reverse transcriptase and random primers. RNA templates were removed and a replacement strand synthesized incorporating dUTP in place of dTTP to generate ds cDNA. The incorporation of dUTP in second strand synthesis quenches the second strand during amplification as the polymerase used in the assay is not incorporated past this nucleotide. AMPure XP beads (Beckman Coulter, A63881) were then used to separate the ds cDNA from the second strand reaction mix, providing blunt-ended cDNA. A single 'A' nucleotide was added to the 3′ ends of the blunt fragments to prevent them from ligating to one another during the subsequent adaptor ligation reaction, and a corresponding single 'T' nucleotide on the 3′ end of the adaptor provided a complementary overhang for ligating the adaptor to the fragment. Multiple indexing adapters were then ligated to the ends of the ds cDNA to prepare them for hybridization onto a flow cell, before 12 cycles of PCR were used to selectively enrich those DNA fragments that had adaptor molecules on both ends and amplify the amount of DNA in the library suitable for sequencing. After amplification libraries were purified using AMPure XP beads.

Libraries were quantified by fluorometry using the Qubit dsDNA HS assay and assessed for quality and fragment size using the Agilent Bioanalyser with the DNA HS Kit (5067-4626). Sequencing was performed using the NextSeq 500/550 High-Output v2 (150 cycle) Kit (FC-404-2002) on the NextSeq 550 platform (Illumina Inc, SY-415-1002). Twenty four libraries were combined in two equimolar pools of 12 based on the library quantification results and each pool was run across a single High-Output Flow Cell. Sequencing was performed at the Wellcome Trust Clinical Research Facility (WTCRF; Edinburgh).

DNA Methylation by Mass Spectrometry

DNA was extracted from frozen cell pellets by standard phenol:chloroform extraction and ethanol purification. To carry out DNA hydrolysis, 2.5 μg DNA in 50 μL final volume was made up to 44μl in mass spectrometry grade water (Chromasolv, Sigma) and incubated at 95°C for 10 mins. 5μl T7 DNA polymerase reaction buffer and 1μl 10U/μl T7 DNA polymerase (Thermo Fisher) were added and the samples incubated o/n at 37°C. The reaction was heat inactivated at 75°C for 10 mins. The sample was then centrifuged at 12,000*g* at r.t. for 45 mins.

Hydrolyzed DNA was extracted in 5:3:2 methanol:acetonitrile:sample, and centrifuged at 12,000*g* for 5 mins, the upper 90 μL were taken and the organic solvent removed using a vacuum centrifuge. Analytes were resuspended in 30 μL mass spectrometry grade water and 10 μL injected onto a 30x 1mm HyperCarb column (VWR). A gradient of 0%–90% B was run over 4 mins, where B is acetonitrile and A is 20 mM ammonium carbonate. Mass spectra were acquired in negative mode on a Thermo Q Exactive, scanning from 300 to 350 m/z at resolution 70k. AGC target was set to 1x 106 and maximum ion time 100ms. Data were analyzed using AssayR (Wills et al., 2017).

In Situ Hi-C

Hi-C was performed largely as described (Rao et al., 2014) with minor modifications. Briefly, 2-5x10^6 mESCs were crosslinked in 1% formaldehyde for 10 mins, snap-frozen and stored at −80°C. After permeabilization in lysis buffer (0.2% Igepal, 10 mM Tris-HCl pH 8.0, 10 mM NaCl, 1x Halt Protease inhibitor cocktail) nuclei were isolated in 0.3% SDS in NEBuffer 3 at 62°C for 10 min. SDS was quenched with 1% Triton X-100 at 37°C for 1 h, then the nuclei were pelleted and resuspended in 250 μl DpnII buffer with 600 U DpnII. After digestion o/n, 200 more units were added for 2 h. Then the ends were filled-in using Klenow, d(G/C/T)TPs and biotin-14-dATP for 1.5 h at 37°C. After ligation at room temperature for 4 h the nuclei were spun down, resuspended in 200 μl mQ and digested with proteinase K for 30 min at 55°C in presence of 1% SDS. Cross-links were reversed at 65°C o/n after addition of NaCl to a final concentration of 1.85 M. After ethanol precipitation and a 70%–80% ethanol wash, DNA was resuspended in 500 μl of sonication buffer (50 mM Tris pH 8.0, 0.1% SDS, 10 mM EDTA), incubated on ice for 15 min and then sheared using a probe sonicator to fragment sizes of 200-700 bp. DNA was then concentrated on Amicon filter units, bound to MyOne T1 Streptavidin beads and used for Illumina library preparation. Small aliquots were taken before and after DpnII treatment, and before sonication to confirm efficient DNA digestion and ligation by running them on 1% agarose gel. Samples were first test-sequenced on NextSeq 550 (WTCRF, Edinburgh) to check library quality, and then selected libraries were sequenced at greater depth on HiSeq 4000 (BGI-Hongkong) (Table S4).

QUANTIFICATION AND STATISTICAL ANALYSIS

FISH Image Analysis

Volocity software (PerkinElmer) was used to capture, process, and analyze the images. Images were deconvolved using the Restoration module, using the constrained iterative algorithm. Image analysis was carried out using the Quantitation module. For analysis of data from ESCs, each dataset consisted of 70-155 measurements. For analysis of Hox probes in blastocysts, 686 alleles from 14 embryos were analyzed. Control inter-probe distances were measured from 100 alleles from 2 blastocysts. The statistical analysis of inter-probe distance distributions was determined using a Mann-Whitney U Test. Mean inter-probe distances for all FISH data are shown in Table S1 and p values are listed in Table S2.

Hi-C Data Analysis

Reads were processed using distiller (https://github.com/mirnylab/distiller-nf) on the high-performance computing cluster of the University of Edinburgh (Eddie). Mapping was performed to the mm9 genome build (Table S4). Hi-C pairs with exactly matching coordinates were removed as PCR or optical duplicates (pcr_dups_max_mismatch_bp: 0). Pairs with mapq < 30 were not used. The output statistics information and Cooler files (https://github.com/mirnylab/cooler) were used in downstream analyses (Abdennur and Mirny, 2019). 1000 bp resolution Cooler files were used to create multi-resolution files for visualization in HiGlass. We only used balanced matrices for our analyses.

We performed pileup analysis using coolpup.py (Flyamer et al., 2019). Briefly, we took all regions of interest in the Hi-C maps, e.g., all *cis* interactions between CGIs bound or not bound by RING1B (Illingworth et al., 2015), and averaged a 205 kb × 205 kb window centered on them at 5 kb resolution. For each averaged window, we also created matrix of expected values, based on average balanced value at each diagonal of the matrix for the same chromosomes. We then summed up all expected matrices and divided observed values by them. Values of enrichment in top left corners of pileups are the enrichment of interactions in the center pixel of the matrix, after all described normalization procedures.

Since our own Hi-C data were not deep enough to call loops with high quality, we chose instead to take advantage of very deeply sequenced published data from mESCs (Bonev et al., 2017).We used *cooltools call-dots* reimplementation of the HiCCUPS algorithm (Rao et al., 2014) from *dekkerlab/shrink-donut-dotfinder* (commit 377106e). This was applied with default settings (except for lower FDR threshold of 0.1) to reanalyzed mapq ≥ 30 filtered mESC Hi-C data at 5 kb, 10 kb and 25 kb resolution to find areas of local enrichment of interactions between loci up to 20 Mb away. Calls from different resolutions were combined using a custom script following the HiCCUPS merging procedure. Annotated dots were then filtered by intersecting with published CTCF peaks (Bonev et al., 2017), and/or RING1B peaks (Illingworth et al., 2015) using *bedtools pairtobed* after widening the peaks using *bedtools slop*.

For local interaction density analysis, we used 5 kb resolution data and 25 kb windows. For each window we determined average observed/expected number of interactions (excluding the first two diagonals, so we averaged 6 pixels per window). If at least 20% of the pixels in the window were missing (NaN), we did not consider it (i.e., > 1 pixel, however since missing values come from masking whole genomic bins during balancing, effectively having one masked bin removed the window from analysis). Then these data were combined with the read coverage in the same windows from ChIP-seq experiments (H3K27me3 from Marks et al., 2012, RING1B from Joshi et al., 2015). Binning of the windows into groups was performed based in quantiles of ChIP-seq values and mean

(±95% confidence interval obtained by bootstrapping) was plotted using *seaborn python* package, together with the total number of windows considered in the analysis after all filtering.

For insulation score analysis, we applied *cooltools diamond-insulation* to data at 25 kb resolution with window size of 100 kb. For eigenvector analysis, we applied *cooltools call-compartments* to data at 200 kb resolution with GC content as the reference track. We then discarded any invalid bins, and performed clustering using *seaborn* package with default parameters, and pairwise Pearson correlation analysis (for insulation) between individual samples to assess the similarity between the samples.

RNA-Seq Analysis

mRNA abundance was quantified using Sailfish (version: 0.9.2,-l ISR) against mm10 transcript models as defined by RefSeq. The R package tximport was used to import and summarize transcript-level estimates for gene-level analysis. The regularized log transformation (rlog, R Package DESeq2) was applied to minimizes differences between samples for rows with small counts, and which normalizes with respect to library size. To visualize sample-to-sample distances a principal component analysis (PCA) was performed using the rlog values.

DATA AND CODE AVAILABILITY

The genomic datasets generated during this study are available at the NCBI Gene Expression Omnibus (https://www.ncbi.nlm.nih.gov/geo).

The accession numbers for H3K27me3 ChIP-seq data for 3B3L cells in serum and 2i are: GSE 72555 (GSM2700276 and GSM2700277).

The accession number for Hi-C data is GSE124342. RNA-Seq data can be accessed using series accession number GSE121171.

The code used to perform pile-up analysis of Hi-C data is available at: https://github.com/Phlya/coolpuppy (Flyamer et al., 2019).

Cell Reports, Volume *29*

Supplemental Information

DNA Methylation Directs Polycomb-Dependent

3D Genome Re-organization in Naive Pluripotency

Katy McLaughlin, Ilya M. Flyamer, John P. Thomson, Heidi K. Mjoseng, Ruchi Shukla, Iain Williamson, Graeme R. Grimes, Robert S. Illingworth, Ian R. Adams, Sari Pennings, Richard R. Meehan, and Wendy A. Bickmore

Supplemental Data

Table S1. Inter-probe distances. Related to Figs 1, 2, S1 and S4

Median inter-probe distances for each FISH probe set in all cell lines and conditions. Data from replicate experiments are indicated

Cell line/condition	Probe pair	Median interprobe distance (µm)
**Figures 1 & S1**		
WT (clone36) Serum	*Hoxd3-Hoxd13*	Rep1: 0.276, Rep2: 0.317
WT (clone36) 2i	*Hoxd3-Hoxd13*	Rep1: 0.424, Rep2: 0.423
WT (clone36) Serum	*GCR-Lnp*	Rep1: 0.334, Rep2: 0.36
WT (clone36) 2i	*GCR-Lnp*	Rep1: 0.36, Rep2: 0.422
Ring1B$^{-/-}$ Serum	*Hoxd3-Hoxd13*	Rep1: 0.379, Rep2: 0.443
Ring1B$^{-/-}$ 2i	*Hoxd3-Hoxd13*	Rep1: 0.483, Rep2: 0.459
Ring1B$^{-/-}$ Serum	*GCR-Lnp*	Rep1: 0.3, Rep2: 0.36
Ring1B$^{-/-}$ 2i	*GCR-Lnp*	Rep1: 0.3, Rep2: 0.334
Eed$^{-/-}$ Serum	*Hoxd3-Hoxd13*	Rep1: 0.483, Rep2: 0.481
Eed$^{-/-}$ 2i	*Hoxd3-Hoxd13*	Rep1: 0.39, Rep2: 0.469
Eed$^{-/-}$ Serum	*GCR-Lnp*	Rep1: 0.334, Rep2: 0.334
Eed$^{-/-}$ 2i	*GCR-Lnp*	Rep1: 0.36, Rep2: 0.334
E14 Serum	*Hoxb1-Hoxb13*	0.276
E14 2i	*Hoxb1-Hoxb13*	0.347
E14 Serum	*Hoxc4-Hoxc13*	0.3
E14 2i	*Hoxc4-Hoxc13*	0.36
**Figure 2**		
Blastocysts	*Hoxd3-Hoxd13*	0.422
Blastocysts	*GCR-Lnp*	0.334
**Figure 5 & S4**		
WT J1 Serum	*Hoxd3-Hoxd13*	0.3
WT J1 2i	*Hoxd3-Hoxd13*	0.443
WT J1 Serum	*GCR-Lnp*	0.36
WT J1 2i	*GCR-Lnp*	0.334
3B3L Serum	*Hoxd3-Hoxd13*	Rep1: 0.3, Rep2: 0.3
3B3L 2i	*Hoxd3-Hoxd13*	Rep1: 0.334, Rep2: 0.334
3B3L Serum	*GCR-Lnp*	0.3
3B3L 2i	*GCR-Lnp*	0.334
3A3L Serum	*Hoxd3-Hoxd13*	0.3
3A3L 2i	*Hoxd3-Hoxd13*	0.36
WT J1 Serum	*En2-Shh*	0.324
WT J1 2i	*En2-Shh*	0.385
WT J1 Serum	*Shh-Mnx1*	0.48
WT J1 2i	*Shh-Mnx1*	0.608
WT J1 Serum	*En2-Mnx1*	0.478
WT J1 2i	*En2-Mnx1*	0.59
3B3L Serum	*En2-Shh*	0.329
3B3L 2i	*En2-Shh*	0.247
3B3L Serum	*Shh-Mnx1*	0.44
3B3L 2i	*Shh-Mnx1*	0.44
3B3L Serum	*En2-Mnx1*	0.471
3B3L 2i	*En2-Mnx1*	0.466

Table S2. Related to Figs 1, 2, 5, S1 and S4

Probability values calculated by Mann-Whitney U tests comparing inter-probe distances between two populations. Inter-probe distances of *HoxD*, *Lnp-GCR* (Ctrl), *HoxC*, and *HoxB* probes in different cell types and conditions are shown

Genotype/ Condition/ Probes Sample 1	Genotype/ Condition/ Probes Sample 2	P-value (Mann-Whitney U Test)
**Figure 1 & Figure S1**		
WT (clone36) Serum HoxD	WT (clone36) 2i *HoxD*	Rep1: <0.0001, Rep2: 0.0003
WT (clone36) Serum HoxD	Ring1B $^{-/-}$ Serum *HoxD*	Rep1: <0.0001, Rep2: <0.0001
WT (clone36) Serum HoxD	Eed $^{-/-}$ Serum *HoxD*	Rep1: <0.0001, Rep2: <0.0001
WT (clone36) Serum HoxD	Ring1B $^{-/-}$ 2i *HoxD*	Rep1: <0.0001, Rep2: <0.0001
WT (clone36) Serum HoxD	Eed $^{-/-}$ 2i *HoxD*	Rep1: <0.0001, Rep2: <0.0001
WT (clone36) Serum Ctrl	WT (clone36) 2i Ctrl	Rep1: 0.4215, Rep2: 0.2564
WT (clone36) Serum Ctrl	Ring1B $^{-/-}$ Serum Ctrl	Rep1: 0.1352, Rep2: 0.5583
WT (clone36) Serum Ctrl	Eed $^{-/-}$ Serum Ctrl	Rep1: 0.7539, Rep2: 0.4776
WT (clone36) Serum Ctrl	Ring1B $^{-/-}$ 2i Ctrl	Rep1: 0.0797, Rep2: 0.1836
WT (clone36) Serum Ctrl	Eed $^{-/-}$ 2i Ctrl	Rep1: 0.5062, Rep2: 0.0865
E14 Serum *HoxB*	E14 2i *HoxB*	0.0334
E14 Serum *HoxC*	E14 2i *HoxC*	0.0024
**Figure 2**		
WT serum *HoxD*	Blastocysts *HoxD* (all)	<0.0001
WT serum *HoxD*	Blastocyst 1 *HoxD*	0.0050
WT serum *HoxD*	Blastocyst 2 *HoxD*	0.0170
WT serum *HoxD*	Blastocyst 3 *HoxD*	0.0168
WT serum *HoxD*	Blastocyst 4 *HoxD*	0.0043
WT serum *HoxD*	Blastocyst 5 *HoxD*	<0.0001
WT serum *HoxD*	Blastocyst 6 *HoxD*	<0.0001
WT serum *HoxD*	Blastocyst 7 *HoxD*	0.0068
WT serum *HoxD*	Blastocyst 8 *HoxD*	0.0002
WT serum *HoxD*	Blastocyst 9 *HoxD*	0.0019
WT serum *HoxD*	Blastocyst 10 *HoxD*	0.1389
WT serum *HoxD*	Blastocyst 11 *HoxD*	0.0537
WT serum *HoxD*	Blastocyst 12 *HoxD*	0.0047
WT serum *HoxD*	Blastocyst 13 *HoxD*	0.0237
WT 2i *HoxD*	Blastocysts *HoxD* (all)	0.0879
WT Serum Ctrl	Blastocysts Ctrl	0.6855
WT 2i Ctrl	Blastocysts Ctrl	0.2017
**Figure 5 and Figure S4**		
WT J1 Serum *HoxD*	WT J1 2i *HoxD*	Rep1: <0.0001, Rep2:
WT J1 Serum *HoxD*	3B3L Serum *HoxD*	Rep1: 0.2027, Rep2:
WT J1 Serum *HoxD*	3B3L 2i *HoxD*	Rep1: 0.2790, Rep2:
3B3L Serum *HoxD*	3B3L 2i *HoxD*	Rep1: 0.8658 Rep2:
WT J1 Serum *HoxD*	3A3L Serum *HoxD*	0.7219
WT J1 Serum *HoxD*	3A3L 2i *HoxD*	0.1128
3A3L Serum *HoxD*	3A3L 2i *HoxD*	0.2779
WT J1 Serum Ctrl	WT J1 2i Ctrl	0.2195
WT J1 Serum Ctrl	3B3L Serum Ctrl	0.0513
WT J1 Serum Ctrl	3B3L 2i Ctrl	0.1445
3B3L Serum Ctrl	3B3L 2i Ctrl	0.5587
WT J1 Serum *Shh-Mnx1*	WT J1 2i *Shh-Mnx1*	0.0001
WT J1 Serum *Shh-Mnx1*	3B3L Serum *Shh-Mnx1*	0.2274
WT J1 2i *Shh-Mnx1*	3B3L 2i *Shh-Mnx1*	<0.0001

3B3L Serum *Shh-Mnx1*	3B3L 2i *Shh-Mnx1*	0.3214
WT J1 Serum *En2-Shh*	WT J1 2i *En2-Shh*	0.0228
WT J1 Serum *En2-Shh*	3B3L Serum *En2-Shh*	0.4785
WT J1 2i *En2-Shh*	3B3L 2i *En2-Shh*	<0.0001
3B3L Serum *En2-Shh*	3B3L 2i *En2-Shh*	0.0028
WT J1 Serum *En2-Mnx1*	WT J1 2i *En2-Mnx1*	0.0101
WT J1 Serum *En2-Mnx1*	3B3L Serum *En2-Mnx1*	0.3550
WT J1 2i *En2-Mnx1*	3B3L 2i *En2-Mnx1*	0.0001
3B3L Serum *En2-Mnx1*	3B3L 2i *En2-Mnx1*	0.3190

Table S3. Details of FISH probes. Related to STAR methods

Genome co-ordinates are given using the mm9 assembly of the mouse genome

Locus	Whitehead Name	Coordinates (mm9)	Size (bp)
Hoxd3	WI1-121N10	Chr2: 74,566,983 – 74,605,438	38,455
Hoxd13	WI1-469P2	Chr2: 74,474,157 -74,513,003	38,846
GCR	WI1-2157A11	Chr2: 74,242,615 -74,282,044	39,429
Lnp	WI1-482L15	Chr2: 74,329,582 -74,372,986	43,404
Hoxb1	WI1-2671L18	Chr11: 96,201,164- 96,242,956	41,793
Hoxb13	WI1-1356F15	Chr11: 96,060,900-96,099,631	38,732
Hoxc4	WI1-0991J24	Chr15: 103,018,285 -103,057,123	38,838
Hoxc13	WI1-1176M4	Chr15: 102,910,943 - 102,949,345	38,403
En2	WI1-2728F4	Chr5: 28,477,913 – 28,517,563	39,650
Shh	WI1-574O18	Chr5: 28,754,458 – 28,795,879	41,421
Mnx1	WI1-1204B6	Chr5: 29,791,124 – 29,827,491	36,367

Table S4. Details of Hi-C reads. Related to STAR methods

Details of unmapped and mapped Hi-C sequence reads and cis vs trans contacts for biological replicates of serum and 2i ESCs.

	serum-1	2i-1	serum-2	2i-2	*serum (1+2)*	2i (1+2)
total	395761788	705371075	583052986	463486298	*978814774*	1168857373
total_unmapped	34422169	61538050	47293653	51315320	*81715822*	112853370
total_singlesided_mapped	84677482	119074965	113483585	97305872	*198161067*	216380837
total_mapped	276662137	524758060	422275748	314865106	*698937885*	839623166
total_dups	153386711	88829285	81632665	73187613	*235019376*	162016898
total_nodups	123275426	435928775	340643083	241677493	*463918509*	677606268
cis	106081324	352699387	293259179	209065335	*399340503*	561764722
trans	17194102	83229388	47383904	32612158	*64578006*	115841546

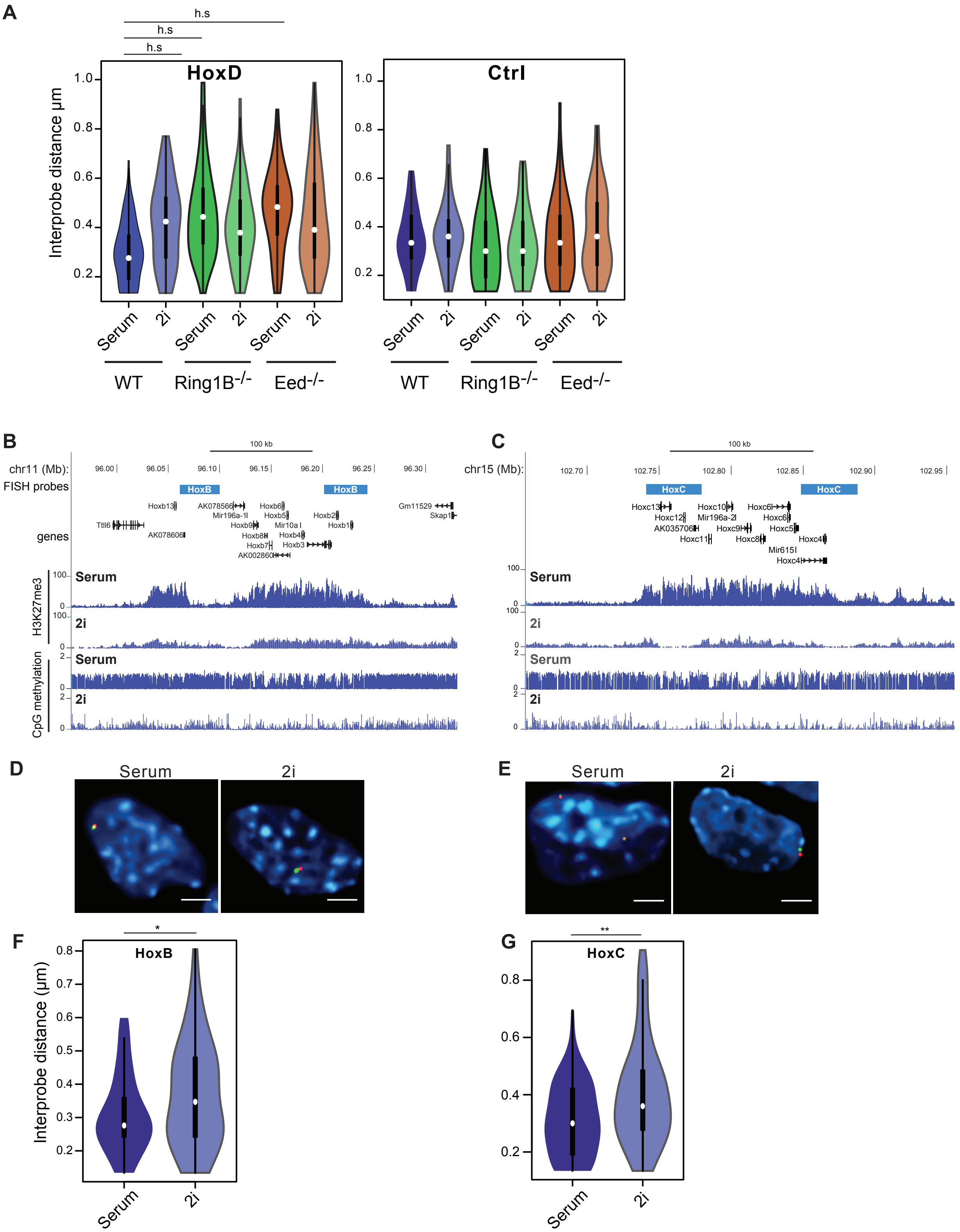

Figure S1. Related to Figure 1.

A. Violin plots showing the distribution of inter-probe distances for HoxD and control (Ctrl) loci (from Figure 1A) in WT, Ring1B-/- and Eed-/- cells grown in serum or 2i. Data are a biological replicate for the data in Figure 1. h.s = $p<0.0001$. Details of statistical analysis are given in Tables S1 and S2.

B. UCSC genome browser tracks (mm9 assembly of the mouse genome) showing the location on chromosome 11 of FISH probes used to measure compaction across the HoxB locus. Probe co-ordinates are given in Table S3. Below are shown the H3K27me3 (Marks et al., 2012) and DNA methylation (Habibi et al., 2013) profiles for this region of the mouse genome in mESCs grown in serum or 2i.

C. As in (B) but for the HoxC locus on chromosome 15.

D. Representative images of HoxB probe hybridisation signals (red and green) in WT E14 mESCs grown in serum or 2i. Scale bars represent 10 µm.

E. As in (D) but for HoxC.

F. Violin plots showing the distribution of inter-probe distances for the HoxB locus in mESCs cells grown in serum or 2i. The vertical line and spot within each plot indicate the interquartile range and median, respectively. *$p<0.05$, **$p<0.01$.

G. As in (F) but for HoxC.

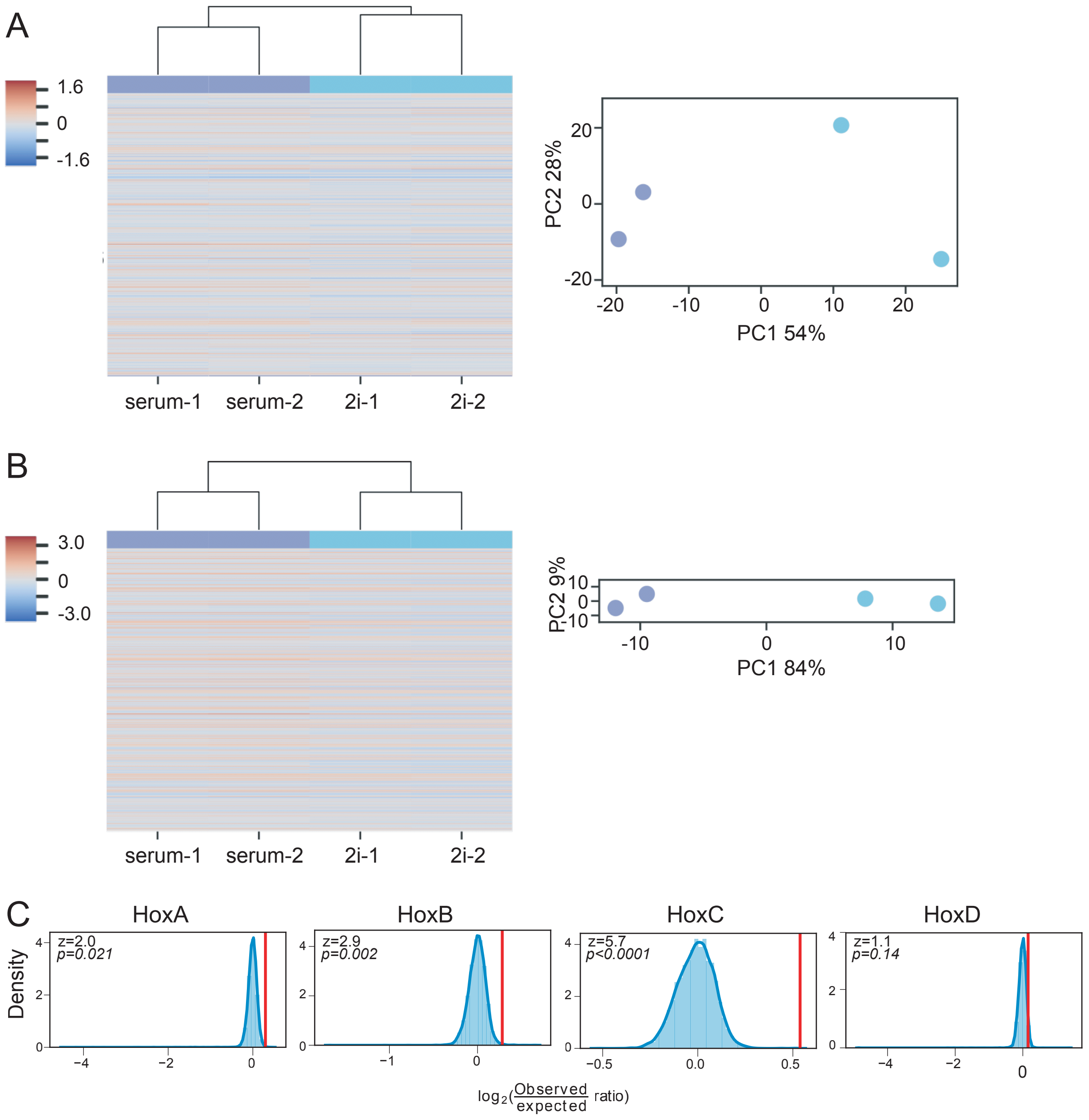

Figure S2. Related to Figure 3.

A. Hierarchical clustering of genome-wide (A) insulation index [25 kb resolution, 1Mb window size] and (**B**) compartment signal/ first eigenvector [200 kb resolution] of Hi-C data from ESCs cultured in serum (purple) or 2i (turquoise). Graphs to the right show principle component analysis of these data.

C. Statistical analysis of the change in number of Hi-C contacts within the four Hox loci. Shown are the distribution of log2(observed/expected) ratios between serum and 2i for 1000 random regions of the same size in the same chromosome as the respective Hox locus; the value for the Hox region is shown with a vertical red line. Z-score and level of significance (p value) are shown in top left corner of each grap

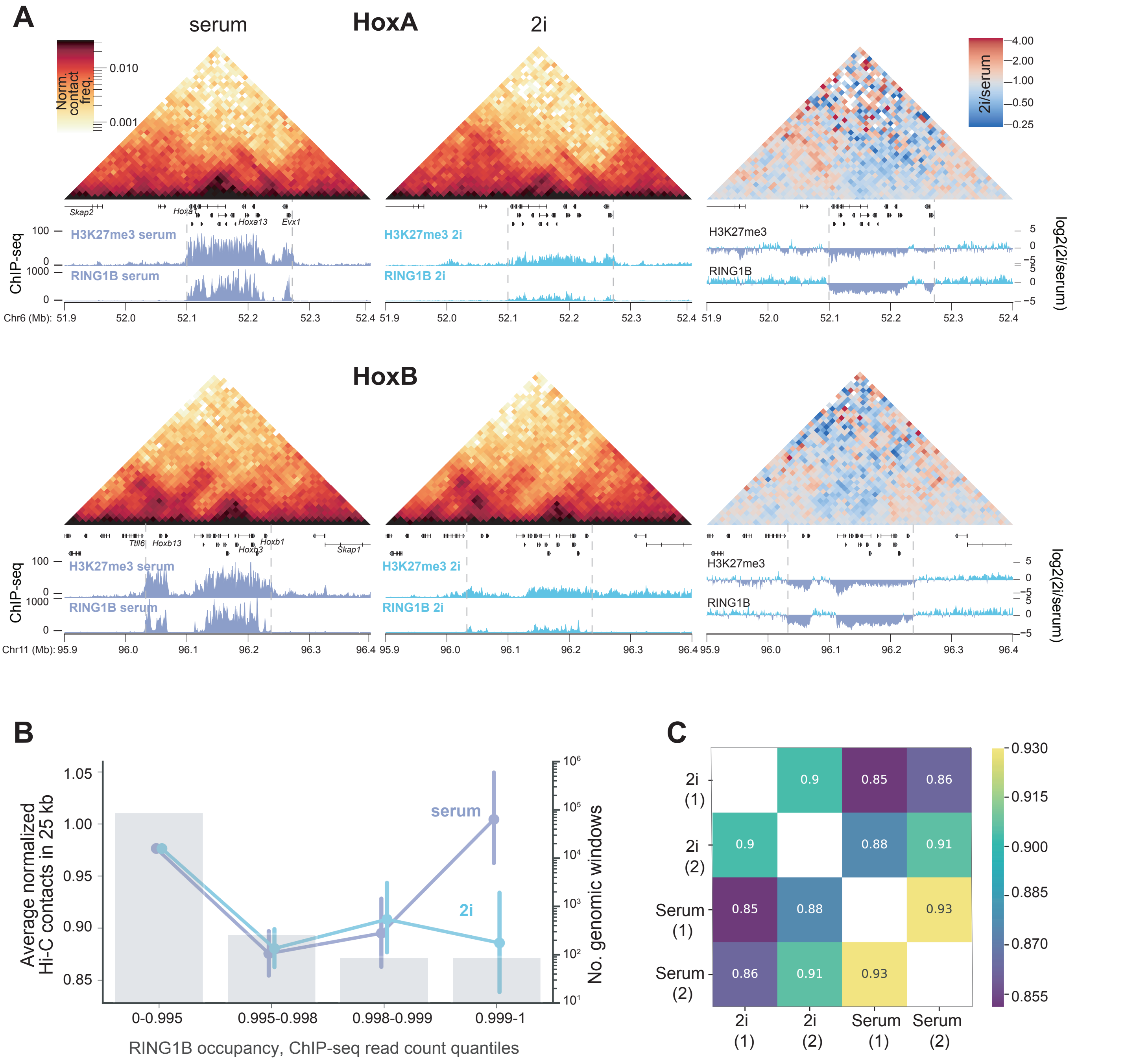

Figure S3. Related to Figure 3.

A. Hi-C heatmaps (normalised contact frequencies) for cells grown in serum (left) and 2i media (middle) for the HoxA (top) and HoxB (bottom). The right hand heatmaps show the difference between contact frequencies in 2i vs serum. Below the gene annotations ChIP-seq profiles for H3K27me3 (Marks et al., 2012), and Ring1B (Joshi et al., 2015) are shown. Boundaries of the Hox clusters are marked with dashed lines. Genome co-ordinates are from mm9 assembly of the mouse genome

B. Same as Fig. 3C, but with RING1B ChIP-seq quantification instead of H3K27me3 (both Hi-C data compared to RING1B data from serum-grown cells). Mean ± 95% CI number of normalized local Hi-C interactions (left hand y axis) in 25 kbp windows across quantiles of RING1B occupancy (Joshi et al., 2015) in serum. Data for serum and 2i media are shown as purple or blue dots respectively. Grey bars show the number of windows in each category (right y axis with log scale).

C. Correlation of log2 of insulation score profiles (100 kb window) across serum and 2i Hi-C replicates.

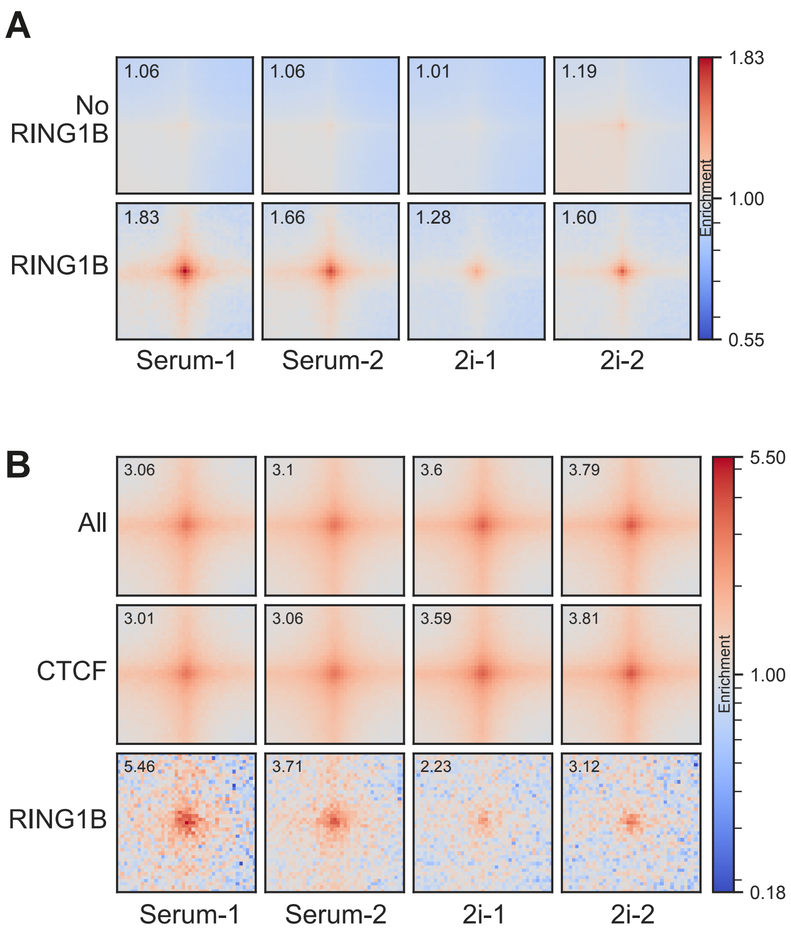

Figure S4. Related to Figure 4.
A. As for main Figure 4C, but for individual replicates.
B. As for main Figure 4D, but for individual replicates of our data.

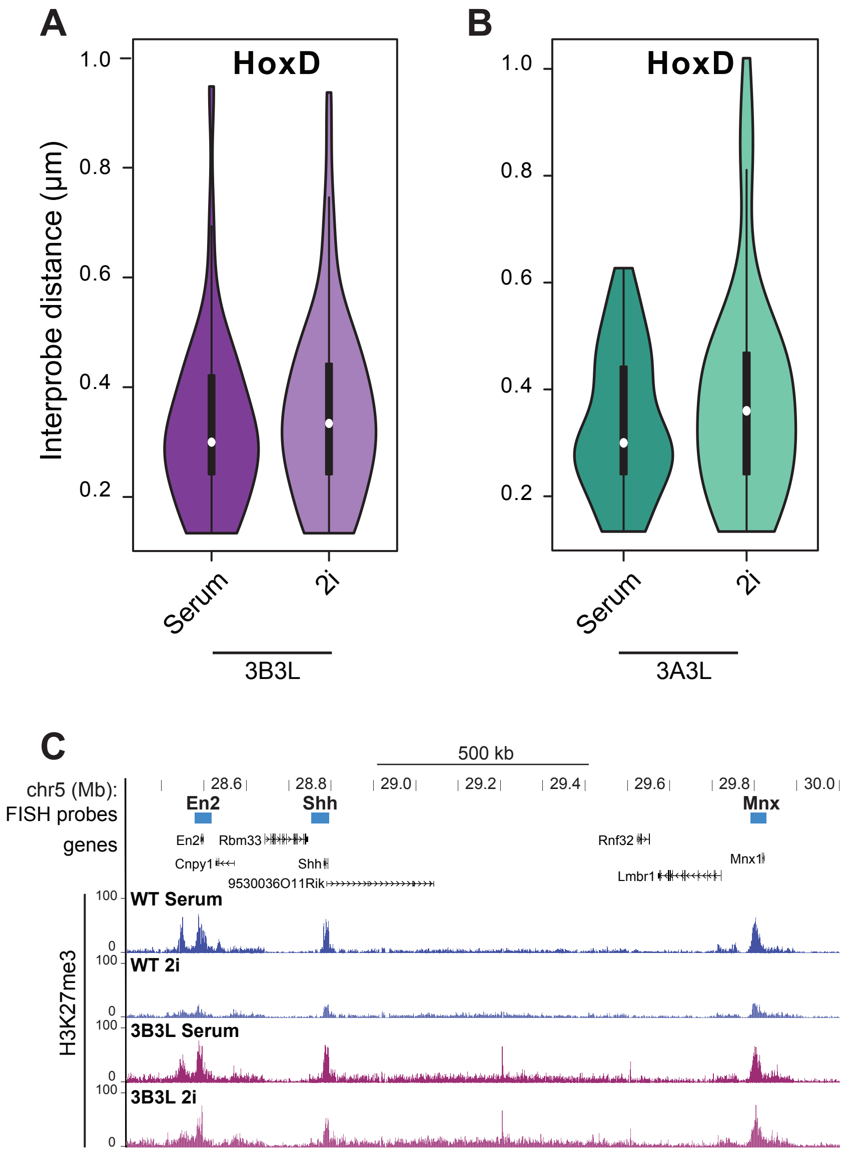

Figure S5. Related to Figure 5.

A. Violin plots showing distribution of inter-probe distances at the HoxD locus in 3B3L cells cultured in serum/LIF and 2i/LIF. This is a biological replicate for the data in Figure 5E.

B. As for (A) but for 3A3L cells.

C. UCSC genome browser tracks (mm9 assembly) showing the location on chromosome 5 of FISH probes used to measure distal interactions across the Shh locus. Probe co-ordinates are given in Table S3. Below are shown the H3K27me3 profiles for this region of the mouse genome in WT (Marks et al., 2012) and 3B3L mESCs grown in serum or 2i.
